# Graphdiyne-based metal atomic catalysts for synthesizing ammonia

**DOI:** 10.1093/nsr/nwaa213

**Published:** 2020-08-28

**Authors:** Huidi Yu, Yurui Xue, Lan Hui, Chao Zhang, Yan Fang, Yuxin Liu, Xi Chen, Danyan Zhang, Bolong Huang, Yuliang Li

**Affiliations:** Institute of Chemistry, Chinese Academy of Sciences, Beijing 100190, China; Institute of Chemistry, Chinese Academy of Sciences, Beijing 100190, China; Science Center for Material Creation and Energy Conversion, School of Chemistry and Chemical Engineering, Institute of Frontier and Interdisciplinary Science, Shandong University, Jinan 250100, China; Institute of Chemistry, Chinese Academy of Sciences, Beijing 100190, China; Institute of Chemistry, Chinese Academy of Sciences, Beijing 100190, China; Institute of Chemistry, Chinese Academy of Sciences, Beijing 100190, China; Institute of Chemistry, Chinese Academy of Sciences, Beijing 100190, China; Institute of Chemistry, Chinese Academy of Sciences, Beijing 100190, China; Institute of Chemistry, Chinese Academy of Sciences, Beijing 100190, China; Department of Applied Biology and Chemical Technology, The Hong Kong Polytechnic University, Hong Kong, China; Institute of Chemistry, Chinese Academy of Sciences, Beijing 100190, China; University of Chinese Academy of Sciences, Beijing 100049, China

**Keywords:** graphdiyne, atomic catalyst, two-dimensional carbon material, ammonia, nitrogen reduction reaction

## Abstract

Development of novel catalysts for nitrogen reduction at ambient pressures and temperatures with ultrahigh ammonia (NH_3_) yield and selectivity is challenging. In this work, an atomic catalyst with separated Pd atoms on graphdiyne (Pd-GDY) was synthesized, which shows fascinating electrocatalytic properties for nitrogen reduction. The catalyst has the highest average NH_3_ yield of 4.45 ± 0.30 mg_NH3_ mg_Pd_^−1^ h^−1^, almost tens of orders larger than for previously reported catalysts, and 100% reaction selectivity in neutral media. Pd-GDY exhibits almost no decreases in NH_3_ yield and Faradaic efficiency. Density functional theory calculations show that the reaction pathway prefers to perform at the (Pd, C1, C2) active area because of the strongly coupled (Pd, C1, C2), which elevates the selectivity via enhanced electron transfer. By adjusting the *p–d* coupling accurately, reduction of self-activated nitrogen is promoted by anchoring atom selection, and side effects are minimized.

## INTRODUCTION

Ammonia (NH_3_), an indispensable raw material in industrial production, has always occupied an important position in basic chemical industry, and is also an important source of chemical fertilizer in agricultural production [1–3]. Unfortunately, traditional production of NH_3_ must operate at high pressures and temperatures, which is very energy-consuming. Electrochemical catalytic nitrogen reduction reaction (ECNRR) in aqueous electrolytes at ambient conditions provides an ideal method for clean and efficient conversion of N_2_ to energy-rich NH_3_ [4–7]. However, there are some key scientific and technical issues with this approach, and the NH_3_ yield (Y_NH3_) and Faradaic efficiency (FE) are still very low. To solve these major issues and promote efficient conversion of N_2_ to NH_3_, efficient catalysts that can drive ECNRR at low overpotential with high selectivity, Y_NH3_, FE and stability are in great demand.

Electrocatalysts comprising singly-dispersed metal atoms and supporting materials have shown high catalytic activity and selectivity in various reactions, because of their atomically distributed active sites [8–21]. Although the rapid development of single-atom catalysts has introduced many new opportunities in terms of new catalytic science, knowledge and concepts, there remain some important scientific issues to be solved, such as the precise structure of the support materials, the valence of the supported metal atoms and the structure, the high dispersion distribution of the metal single atoms and so on.

Very recently, Li and coworkers reported the first zero-valent atomic catalysts (AC) [20,21]. An important finding that has drawn great attention is that zero-valent metal atoms can be anchored on graphdiyne surfaces [22–31]. The AC exhibits high stability and catalytic activity and represents the emergence of a new generation of catalysts, and there is a drive to understand the mechanism, reaction process and properties of atomic catalysis. In this study, we demonstrate that highly selective and active NH_3_ production can be achieved using a zero-valence atom catalyst based on graphdiyne, Pd-GDY, to activate the N_2_ and react with water at room temperature and pressure. Experimental and theoretical results solidly confirm the unique chemical and electronic structures and zero-valence state of this electrocatalyst. The strong orbital interactions between Pd atoms and neighboring C sites lead to a strong electronegative reduction character for ECNRR. The downshifted s-band from the electronic structure arises from the elimination of non-bonding lone-pair N2-2s orbitals through N-hydrogenation to suppress HER electronically. The designed Pd-GDY AC presents significantly improved ECNRR performance compared with previously reported catalysts. This study could provide a promising strategy for designing and synthesizing highly efficient electrocatalysts for producing NH_3_ at room temperature and ambient pressures.

## RESULTS AND DISCUSSION

**Figure 1.**
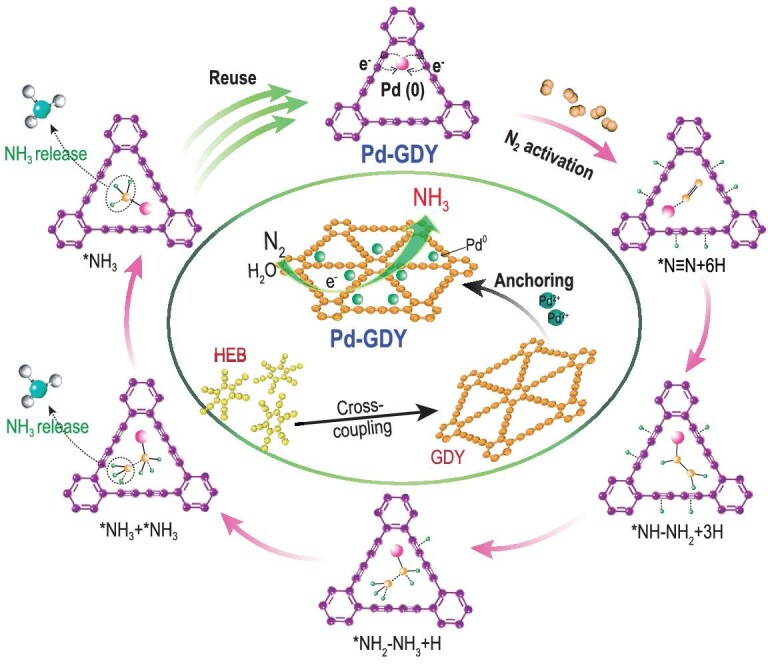
Schematic of the synthesis (central green circle) and reusability of the Pd-GDY electrocatalyst for the ammonia production.

Pd-GDY was synthesized through a self-reduction strategy. Figure 1 shows the synthesis and reusability schematic of Pd-GDY for the electrochemical nitrogen reduction reaction. As can be seen from the scanning electron microscopy (SEM) images (Supplementary Fig. S1), graphdiyne fibers with porous surface interweave in a three-dimensional manner forming three-dimensional flexible electrodes.

**Figure 2.**
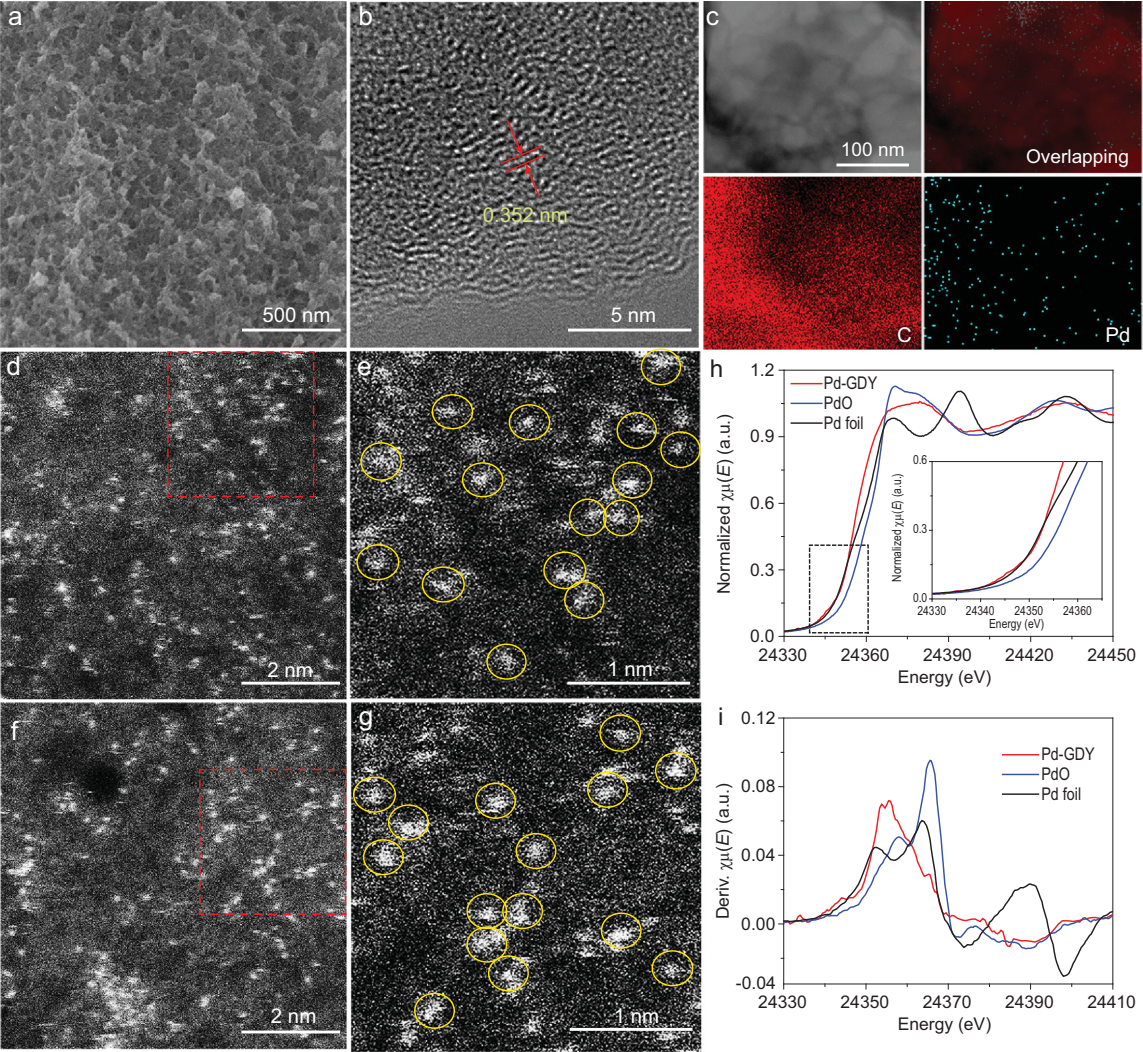
(a) SEM, (b) HRTEM, (c) elemental mapping, and (d and f) HADDF-STEM images of Pd-GDY. (e and g) Enlarged images of the red square areas in images (d) and (f), respectively. (h) The experimental K-edge XANES spectra (inset: magnified image) and (i) the first derivative curves of Pd-GDY, Pd foil and PdO.

No Pd particles or clusters can be observed in the SEM (Fig. 2a and Supplementary Fig. S2), high-resolution transmission electron microscopy (HRTEM) (Fig. 2b and Supplementary Fig. S3), scanning transmission electron microscopy (STEM, Fig. 2c) and high-angle annular dark-field (HAADF) STEM (Fig. 2d–g and Supplementary Fig. S4) images. Energy-dispersive X-ray spectroscopy mapping results (Fig. 2c) reveal the uniform distribution of Pd and C atoms in the sample. In the HAADF images, the bright dots gave an average size of 0.36 ± 0.01 nm (Supplementary Fig. S5), confirming that each dot is an individual Pd atom. The possible anchoring sites of Pd atoms on GDY are shown in Supplementary Fig. S6, as revealed by our detailed computational calculations in which all possible placements of the Pd on GDY were fully examined. The optimal anchoring site is determined based on comparison of formation energies between different anchoring sites, in that the lowest formation energy of the anchoring site of −0.99 eV as the presented position will be the optimal placement for Pd on GDY. Inductively coupled plasma optical emission spectroscopy (ICP-OES) measurements showed an average mass loading of 1.02 ± 0.04 wt.%. Figure 2h and i shows the X-ray absorption near-edge structure (XANES) profiles for Pd-GDY along with corresponding reference samples (Pd foil and PdO). For the Pd K-edge spectrum, the absorption threshold position of Pd-GDY is located at the same position as Pd metal but a more negative position than PdO (Fig. 2h). In addition, the first derivative XANES for Pd-GDY is similar to that of metallic Pd (Fig. 2i). These results strongly indicate that the valence states for the Pd atoms in Pd-GDY metals are zero. The extended X-ray absorption fine structure (EXAFS) spectrometry results (Supplementary Fig. S7) showed that there was only one peak at around 1.5 to 2 Å arising from the Pd∼C contribution, and that no peak contributing to the Pd–Pd (around 2∼3 Å) could be observed. These demonstrate that the Pd atoms are individually anchored on GDY and exhibit zero-valence state (Supplementary Fig. S8).

We used X-ray photoelectron spectroscopy (XPS) and Raman spectra to characterize the chemical composition and quality of as-synthesized samples. The XPS survey spectra show the only existence of carbon (Supplementary Fig. S9), except for oxygen signal from the adsorption of air. The C 1s XPS spectrum of Pd-GDY can be divided into sp^2^-C (284.5 eV), sp-C (285.1 eV), C=O (288.3 eV), COO (286.7 eV) and π–π transition (291.2 eV) peaks (Supplementary Fig. S10a). Compared with pure GDY (Supplementary Fig. S10b), a newly formed peak at 291.2 eV was observed, which can be attributed to the electronic transitions induced by the anchoring of Pd atoms. The ratio of intensity of sp^2^/sp-carbon is 0.5, revealing the integrity of the GDY structure in Pd-GDY. As shown in Supplementary Fig. S11, the Pd-GDY shows a lower valence band (VB) with a smaller energy at 1.73 eV than that of pure GDY (2.26 eV), indicating the enhanced conductivity of Pd-GDY. Raman spectra provide information on the structural changes of carbon materials. The intensity of the D and G bands of GDY and Pd-GDY changed (Supplementary Fig. S12). The D band to G band intensity ratio increased from 0.73 (GDY) to 0.82 (Pd-GDY), which suggests the presence of more defective sites in Pd-GDY, indicating the formation of more active sites, which have been demonstrated to be helpful for improving catalytic activity [32,33].

**Figure 3.**
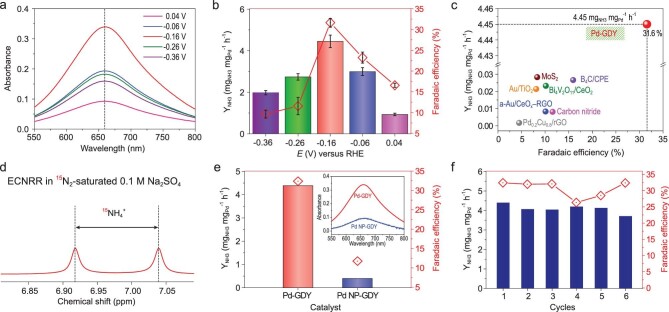
(a) UV–Vis absorption spectra of the 0.1 M Na_2_SO_4_ electrolytes after ECNRR at different potentials for 2 h. (b) Y_NH3_ and FEs at applied potentials in 0.1 M Na_2_SO_4_. Error bars represent calculated standard deviation from independent experiments (at least three times). (c) Comparison of the ECNRR performance of Pd-GDY with others. Error bars represent calculated standard deviation from independent experiments (at least three times). (d) ^1^H-^15^N NMR spectra of 0.1 M Na_2_SO_4_ after ECNRR under ^15^N_2_ with Pd-GDY as catalyst. (e) Y_NH3_, FEs and corresponding UV–Vis absorption spectra (inset) of Pd-GDY and Pd NP/GDY catalysts after 2 h electrolysis at −0.16 V versus RHE. (f) Stability test of Pd-GDY at −0.16 V versus RHE in 0.1 M Na_2_SO_4_ under ambient conditions.

Based on the above inspiring findings, the ECNRR experiments were carried out in 0.1 M Na_2_SO_4_ (pH = 7) aqueous solution at room temperature and atmospheric pressure using an H-type electrolytic cell separated by the Nafion 117 membrane (Supplementary Fig. S13). The catalyst loading of Pd-GDY is 2.7 μg_metal_ cm^−2^, and the geometric area is 2.0 cm^2^. The NH_3_ was determined using an indophenol blue method [34] and calibration curves with reliable sensitivity and good linear relationship were obtained (Supplementary Fig. S14). The ECNRR of Pd-GDY starts at around 0.2 V versus RHE in N_2_-saturated 0.1 M Na_2_SO_4_ solution (Supplementary Fig. S15). Only NH_3_ (no by-product of N_2_H_4_) can be detected in this work (Supplementary Figs S16 and S17), revealing the ultra-high selectivity of Pd-GDY in the ECNRR process. The NH_3_ yielding rate (Y_NH3_) and the Faradaic efficiency (FE) rise with increasing cathodic potential until −0.16 V versus RHE (Fig. 3a and b), at which the highest Y_NH3_ of 4.45 ± 0.30 mg_NH3_ mg_Pd_^−1^ h^−1^ (1.97 ± 0.13 × 10^−^^10^ mol cm^–2^ s^–1^, normalized by geometric surface area) and FE up to 31.62 ± 1.06% were achieved (Supplementary Table S1). These values are larger than for all previously reported ECNRR catalysts working under ambient conditions including Au/TiO_2_ (Y_NH3_ = 21.4 μg_NH3_ mg_cat._^–1^ h^–1^, FE = 8.11%) [6], THH Au NRs (Y_NH3_ = 2.69 × 10^–^^11^ mol cm^–2^ s^–1^, FE = 4.02%) [35], Bi_4_V_2_O_11_/CeO_2_ (Y_NH3_ =23.21 μg_NH3_ mg_cat._^–1^ h^–1^, FE = 10.16%) [36], Ru SAs/N-C (Y_NH3_ = 120 μg_NH3_ mg_cat._^−1^ h^−1^, FE = 29.6%) [37] and Ru NC (Y_NH3_ = 3.6 mg_NH3_ mg_cat._^−1^ h^−1^, FE = 21%) [38], and Pd-based ECNRR catalysts such as Pd/C (Y_NH3_ = 4.9 μg_NH3_ mg_cat._^−1^ h^−1^, FE = 8.2%) [39], Pd_0.2_Cu_0.8_/rGO (Y_NH3_ = 2.8 μg_NH3_ mg_cat._^−1^ h^−1^, FE = 4.5%) [40], Pd_3_Cu_1_ alloy (Y_NH3_ = 39.9 μg_NH3_ mg_cat._^−1^ h^−1^, FE = 1.22%) [41], and even compared favorably with those working at higher temperatures/pressures (Fig. 3c; Supplementary Tables S2 and S3) [42]. With further increase of the negative potentials, the Y_NH3_ and FE decreased sharply, which can be attributed to competition between ECNRR and HER [21,43]. Control experiments were performed to determine the origin of the detected NH_3_. The pristine GDY yielded limited NH_3_ (Y_NH3_: 5.44 × 10^–^^12^ mol cm^–2^ s^–1^; FE: 1.65%; Supplementary Fig. S17a,b) and CC substrate yielded almost no NH_3_ (Supplementary Fig. S17a). No NH_3_ can be detected in Ar-saturated electrolyte (Supplementary Fig. S17c) and at the open-circuit potential (Supplementary Fig. S17d). ^15^N-labelling experiments were performed using ^15^N_2_ as the feeding gas to confirm the ammonium formed from the reduction of N_2_. Only ^15^NH_4_^+^ (doublet peak) was

observed from the ^1^H nuclear magnetic resonance (^1^H-NMR) results (Fig. 3d). These results revealed that all detected NH_3_ comes from reduction of N_2_ molecules by the Pd-GDY catalyst. The fact that the Y_NH3_ and FE of Pd-GDY are almost 10 and 3 times larger than those of palladium nanoparticle-modified GDY (Pd NP/GDY, Supplementary Fig. S18) demonstrates the superiority of isolated zero-valence Pd atoms over bulk Pd nanoparticles toward efficiently catalyzing ECNRR (Fig. 3e). In addition to excellent catalytic activity and selectivity, long-term stability is another essential criterion for an electrocatalyst in practical applications. It was observed that the NH_3_ yield rate and FE of Pd-GDY exhibited no obvious change after six successive catalytic cycles (Fig. 3f, Supplementary Fig. S19), and the total NH_3_ yield increased linearly with reaction time (Supplementary Fig. S20). Comprehensive characterizations (Supplementary Fig. S21) on the sample obtained after stability test showed good preservation of the morphology and structure, revealing its robust nature.

Electrolytes are considered to have significant effects on the ECNRR selectivity and activity [39,43–45]. Neutral and basic electrolytes with limited proton transfer rate can effectively improve the ECNRR selectivity. However, in acidic electrolytes, the ECNRR selectivity could be kinetically limited because of the high availability of protons; the rate of the HER increases significantly with the increase of applied potentials, in which most protons or H_2_O are converted to H_2_ rather than NH_3_, resulting in great decreases in reaction selectivity. Efforts have recently been devoted to improving ECNRR performance in acidic electrolytes [37,44,46–48]. However, the Y_NH3_ and Faradaic efficiencies of reported ECNRR electrocatalysts are still very low (Supplementary Tables S2 and S3). Because of these limitations, it is of significant importance to develop an electrocatalyst with high selectivity and activity in acidic electrolytes for efficient ECNRR.

**Figure 4.**
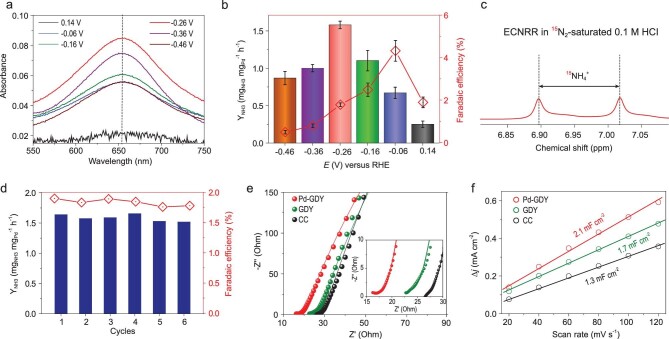
(a) UV–Vis absorption spectra of the 0.1 M HCl electrolytes after ECNRR at different potentials for 2 h. (b) Y_NH3_ and FEs at applied potentials in 0.1 M HCl. (c) ^1^H-^15^N NMR spectra of 0.1 M HCl after ECNRR under ^15^N_2_ with Pd-GDY as catalyst. (d) Stability test of Pd-GDY at −0.26 V versus RHE under ambient conditions. (e) Measured and fitted impedance data of the samples. (f) Plot of current density versus scan rates of 20, 40, 60, 80, 100 and 120 mV s^−1^, respectively.

We then studied the ECNRR activity of Pd-GDY in 0.1 M HCl solution (Fig. 4a, Supplementary Fig. S22). NH_3_ and N_2_H_4_ were determined using a spectrophotometry method, and the calibration curves are shown in Supplementary Fig. S23. Pd-GDY also shows high selectivity toward formation of NH_3_ (without N_2_H_4_ formation) in acidic media (Supplementary Figs S22b and S24). As shown in Fig. 4b, the average NH_3_ yield rate reaches a maximum value of 1.58 ± 0.05 mg_NH3_ mg_Pd_^–1^ h^–1^ [(6.99 ± 0.22) × 10^–^^11^ mol cm^–2^ s^–1^ at −0.26 V versus RHE], while it achieves the highest FE of 4.32 ± 0.49% at a cathodic potential of −0.06 V versus RHE (Supplementary Table S1). The ^15^N isotope labelling experiments confirmed that the NH_3_ was formed from reduction of N_2_ (Fig. 4c), which indicates that the Pd-GDY is highly selective and active

toward ECNRR in acidic media. The FE value keeps decreasing as the applied potentials become more negative, which can be attributed to competition from the HER in acidic media. The rapid increase of current densities in polarization curves recorded in both N_2_- and Ar-saturated 0.1 M HCl (Supplementary Fig. S25) also gives supportive evidence for this major interference. Despite all this, the ECNRR activity of Pd-GDY in acidic conditions still compared favorably with those of most previously reported catalysts (Supplementary Tables S2 and S3) such as MoS_2_ (Y_NH3_ = 8.48^–^^11^ mol cm^–2^ s^–1^, FE = 0.096%). In addition, both Y_NH3_ and FE of Pd-GDY show negligible degradation after six consecutive tests, confirming reliable stability of Pd-GDY in acidic media (Fig. 4d). Electrochemical impedance spectroscopy (EIS) was conducted to offer more insight into the catalytic behaviors. The impedance data were recorded and analyzed using a R(QR)(QR) equivalent circuit model (Supplementary Table S4). From Fig. 4e, Pd-GDY shows the lowest solution resistance (R_s_, 10.41 Ω) and charge transfer resistance (R_ct_, 3501 Ω) over GDY (R_s_, 15.70 Ω; Rct, 4658 Ω) and CC (R_s_, 26.5 Ω; Rct, 8933 Ω), indicating a more kinetically favorable ECNRR for Pd-GDY than others. Electrochemically active surface area (ECSA) was further estimated by determining the electrochemical double-layer capacitance (C_dl_) through a cyclic voltammetry method (Supplementary Fig. S26). The C_dl_ value of Pd-GDY is 2.1 mF cm^–2^ (Fig. 4f), which is larger than those of pure GDY (1.7 mF cm^–2^) and CC (1.3 mF cm^–2^), implying an increase of the active site number in the Pd-GDY catalyst.

**Figure 5.**
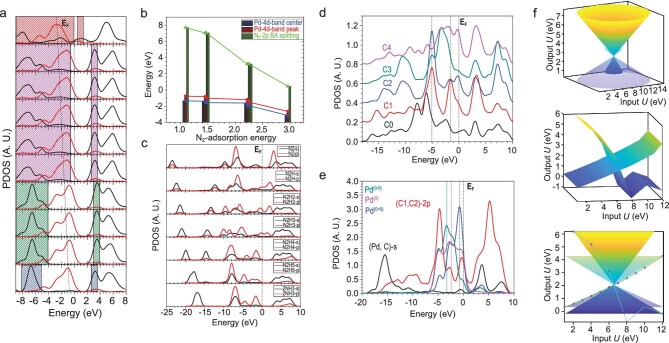
(a) PDOSs evolutions of site-dependent energetic preference N-fixation on the GDY-Pd system. The energetic preference sequence follows the sequence of blue (strongly favorable), green, pink and red (unfavorable) shaded area. (b) The variation behavior of the Pd-4d band center, Pd-4d band peak position and N_2_–2p BA splitting related to the N_2_-adsorption energy. (c) PDOSs of s- and p- bands of all related N-species and adsorbing H among the ECNRR steps. (d) PDOSs of p-orbitals of C0, and C1–C4. The C0 is the C site of benzene ring and C1–C4 are labelled sequentially following the C0 along the C-chain bonding with Pd. (e) PDOSs of s, p and d orbitals from Pd-(C1, C2) bonding motifs from GDY-Pd with consideration of different charge populations. (f) The orbital potential energy projections (*U*_out1_ and *U*_out2_) for Pd-4d within a singly anchoring site under the planar and round constant electrode potential distribution.

The origin of the outstanding catalytic activities of the samples was further studied with computational calculations (see Methods for details). The bonding and anti-bonding orbitals near the Fermi level (E_F_) are demonstrated for the cases of Pd-GDY, HER and ECNRR. We found that the (Pd, C1, C2) sites are electron-rich regions, exhibiting the localized *p–d* coupled electronic orbital. The variation of the real-spatial distribution of charge density distribution indicates site-to-site charge migration and redistribution between the Pd-GDY and N/H-species (Fig. 5a). The interplay of Pd-4d and N_2_–2p orbitals was illustrated with the projected partial density of states (PDOSs). We found that the preference of N_2_-adsorption on the Pd-GDY interacting with Pd-4d orbitals indeed follows the farthest *p–d* separation rather than *p–d* orbital overlapping. This is evidently different from the adsorption preference of O-species. Such an anomalous trend implies that the on-site effective screening Coulomb repulsion potential is a determining factor existing between Pd-4d^10^ and N_2_-(2s^2^, 2p^3^). This arises because the non-bonding lone-pair N_2_–2s-orbitals are highly active, participating within the ECNRR process for hydrogenation bonding. The fulfilled Pd-4d^10^ is an electron-rich center for evident site-to-site charge migrations, exhibiting substantially strong electronegative activity (ENA). Such strong ENA induces high chemical potential contrast for favorite directional electron transfer, which favors electron transfer from Pd onto N-species for N-hydrogenation rather than HER. We further interpret the underlying electronic mechanism to redirect the HER-suppression trend. The *p–d* coupled effective negative correlation energy matters to the subtle interplay between on-site Coulomb repulsion and ENA, which overcomes on-site Coulomb repulsion and is energetically favorable to accumulate ENA (Fig. 5a). The Pd-4d band center, Pd-4d band dominant peak, and splitting gap between bonding and anti-bonding (BA) of N_2_–2p-band all exhibit the same trend to monotonically decrease with N_2_-adsorption energy increases. The deep localized Pd-4d^10^-*t_2g_* component strongly couples with the N_2_–2p band, resulting in a narrowing of the N_2_–2p band BA splitting gap (Fig. 5b). We further demonstrate the s- and p- band variations among the N-hydrogenation process. The downshifted s-band arises from the elimination of non-bonding lone-pair N_2_-2s orbitals through N-hydrogenation. The N-hydrogenation performs 0.8 eV more than the H-adsorption at the C site. Therefore, the original HER performance has been electronically suppressed (Fig. 5c). Considering the contribution of individual C sites within Pd-GDY, both C1 and C2 sites clearly show a strong 2p electronic state at E_V_-5.0 eV as relay-center-like level promoting electron transfer between Pd and (C1, C2). The evident bonding and anti-bonding splitting feature at the C1 site confirms that the strong Pd-C1 interaction is indeed enhanced and further stabilized via charge transfer (Fig. 5d). The PDOSs of Pd-4d and (C1, C2)-2p orbitals presented a large overlap near the E_F_, and the two dominant BA orbitals of (C1, C2) coupled the Pd-4d bands. The metallic fcc-Pd^0^, Pd^0−δ^, and Pd^0+δ^ states (0 < δ < 1) were compared within the PDOSs. The Pd^0−δ^ shows 1 eV lower than the fcc-Pd^0^, while Pd^0+δ^ stays 1.5 eV higher. This reveals that Pd-GDY preserves the Pd^0^ state and possesses an even higher ENA than the fcc-Pd^0^, which dominates the strong electronegative reduction character for ECNRR (Fig. 5e). We further determined the Pd-4d orbital information under the electrode potential of the planar and round constant potential distributions. Strong orbital interaction between Pd and neighboring C sites induces a close shell effect by way of crossover. The crossover orbital energy turns to be the equivalent point for repulsion and ENA energetic competition, which stays at 6.55 eV beyond the original energy found from fcc-Pd (2.51 eV). This implies that the ENA has been promoted by the negative correlation to overcome the orbital repulsion (Fig. 5f).

**Figure 6.**
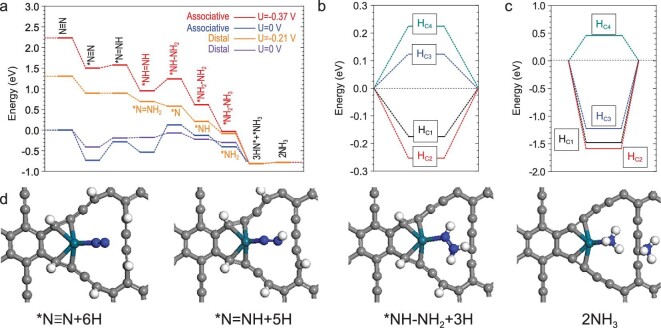
(a) ECNRR energetic pathway on the GDY-Pd. (b) Formation energies of H-adsorption on C sites of GDY-Pd. (c) H-chemisorption energies on C sites of GDY-Pd. (d) Structural configuration evolution of ECNRR catalysis process.

We move on to the energetic preference of ECNRR. Benchmarked from different adsorption configurations, the pathway prefers to perform at the (Pd, C1, C2) active area. This is because the strongly coupled (Pd, C1, C2) elevates the selectivity via enhanced electron transfer, as discussed above. The Pd site dominated preferable pathway was then considered. The Pd-GDY possesses potential of U = −0.37 V for ECNRR and shows a mainly downhill energetic trend. Between these two parts, the Pd site dominates the optimal N-fixation and the C sites along the C-chain distribute the H-adsorption. For U = −0.37 V, the overall energy gain is −3.01 eV, where the NH_3_-desorption is almost barrier-free of merely 0.03 eV for each NH_3_. For U = 0 V, the path confronts the barrier at the asymmetrical N-hydrogenation step until the formation of ^*^NH-NH_2_ + 3(H^+^+e^−^), which starts a downhill trend. This hydrogenation step determines the overall reaction barrier of 0.66 eV. The formation of ^*^N = NH + 5(H^+^+e^−^) also shows an uphill step, but a lower barrier of 0.45 eV. Therefore, the asymmetric H-desorption from the (C1, C2) sites controls the barrier acting as a potential determining step (PDS) for the ECNRR (Fig. 6a). We compared site-dependent H-adsorption energies. The C1 and C2 sites are confirmed as energetically favorable sites for H-adsorption. Further H-chemisorption energies summarize that both C1 and C2 contribute two optimal active sites for H-adsorption for efficient proton-electron charge exchange (Fig. 6b and c). From pathway analysis, we found that the ECNRR prefers parallel hydrogenation (late N–N cleavage) over serial hydrogenation (early N–N cleavage). Further local structural configurations of the ECNRR process demonstrate that late N–N bond dissociation starts at the step of NH_2_−−NH_3_+(H^+^+e^−^), which is advantageous for better HER suppression. This shows a consistent trend that the intermediate nitrogen-nitrogen bonding (i.e. N=N and N–N) variation ensures energetic compensation for H-desorption from the local active adsorption sites (Fig. 6d).

## CONCLUSION

In summary, our experimental and theoretical results strongly support a zero-valence state of the palladium atom that can be anchored on graphdiyne surfaces. The anchoring site of a zero-valence metal atom was observed directly by XANES, and the position of the atomic site as judged by sight is in agreement with the theoretical calculation. The derivative X-ray absorption near-edge structure (XANES) of Pd-GDY is similar to that of metallic Pd, which confirms that the stable valence state of Pd atoms in the sample is zero. The zero-valence Pd atoms separated from graphdiyne show high ECNRR activity and selectivity with the highest N_2_ reduction activity and average yield of NH_3_ at 4.45 ± 0.30 mg_NH3_ mg_Pd_^−1^ h^−1^ under environmental conditions. Our work represents a new concept of catalysis, which is of great significance for a deeper understanding of the catalytic process and mechanism of zero-valence atomic catalysts, especially the discovery of highly selective atomic catalysts for conversion of nitrogen to ammonia at high efficiency.

We note the rapid development of single-atom catalysts in recent years, which has led to the rapid development of catalytic science. Prof. Tao Zhang [8,9,11], Prof. Jun Li [11,49,50] and Prof. Yadong Li [16,17,19] *et al.* have made many contributions to basic and applied research on single-atom catalysts, leading the progress in this field. Our study cleverly uses the advantages of the electronic and chemical structures of graphdiyne to successfully anchor transition metal and noble metal zero-valent atoms on graphdiyne, so we call this an ‘atomic’ catalyst. Loading zero-valent metal atoms has been an important challenge in the field of catalysts, especially for anchoring transition metal atoms. The success of loading zero-valent metal atoms on graphdiyne expands the synthesis methodology of single-atom catalysts and enriches the types of functional catalysts. The birth of zero-valent atomic catalysts is of great academic significance for us to clearly understand the anchoring process of single atoms on the support, the new catalytic process and mechanism, the interaction of metal atoms with the support, the energy, electron transfer and conversion in the system, and the relationship between the catalytic performance and the above scientific content. The emergence of zero-valent catalysts is a real insight into the strategies involved in the development of new single-atom catalysts. The following strategies are the principles of our later design and synthesis of highly efficient catalysts: (i) the design of support is an important basic strategy related to catalyst stability, selectivity and efficiency; (ii) the energy transfer and electron transport between the support and different metal atoms must be considered; (iii) systems theory must be developed for single-atom and atomic catalysts, and how to use machine learning to screen functionalized catalysts; (iv) in particular, it is necessary to develop highly conjugated supports to form donor-acceptor (D–A) systems with metal atoms, understand the mode of interaction of metal atoms and supports, and better understand the regular of structure and performance of the system; and (v) it must be considered how to control the amount of charge transfer in a single-atom system to realize the regulation of catalyst activity.

## METHODS

### Preparation of GDY electrode

GDY electrodes were prepared according to previous studies [20,21,23]. Typically, 50 mL of a pyridinic solution of hexaethynylbenzene (HEB, 0.4 mg mL^−1^) was added very slowly to a three-necked flask containing several pieces of copper foil and carbon cloth (CC) at 110°C. After a 3-day reaction (110°C, protected from air and light), the CC was washed with hot acetone, DMF, KOH (4 M), HCl (6 M), KOH (4 M) and water, sequentially, followed by drying in a 40°C vacuum oven for 12 hours. The GDY electrodes were obtained.

### Preparation of Pd-GDY

A piece of the freshly prepared GDY electrode was immersed into 35 mL H_2_SO_4_ aqueous solution containing 12 mg PdCl_2_. During this process, the Pd atoms could spontaneously anchor to the GDY surface. After a 4-hour reaction, the samples were washed with 0.5 M H_2_SO_4_ and water in sequence, and then immediately used for ECNRR.

### Characterizations

SEM images were obtained from an S-4800 field emission scanning electron microscope. TEM, HRTEM and EDX mapping data were collected using a JEM-2100F electron microscope operating at 200 kV. (HAADF) STEM measurements were conducted on aberration-corrected cubed FET Titan Cubed Themis G2 300 or JEM-ARM200F (JEOL, Tokyo, Japan). XRD patterns were recorded using a D/max-2500 rotation anode X-ray diffractometer (Rigaku, Japan) with Cu Kα radiation (λ = 1.54178 Å). Raman measurements were performed on a Renishaw-2000 Raman spectrometer (473 nm excitation laser source). A Thermo Scientific ESCALab 250Xi instrument with monochromatic Al Kα X-ray radiation was used to perform the XPS measurements.

### ECNRR measurements

ECNRR measurements were carried out on an electrochemical workstation (CHI 660E) with H-type electrolytic cell, which was separated by the Nafion 117 membrane into two chambers. The freshly prepared Pd-GDY (or other reference sample) was used as a working electrode. The catalyst loading of Pd-GDY catalyst is 2.7 μg_metal_ cm^−2^, and the geometric area is 2 cm^2^. The graphite rod and saturated calomel electrode were used as the counter and reference electrodes, respectively. 30 mL N_2_-saturated electrolyte (0.1 M Na_2_SO_4_ or 0.1 M HCl) was added into the chambers. N_2_ flow was continuously fed into the cathodic side with proper position. The catalytic activities of Pd-GDY normalized by geometric area and catalyst loading were compared with other recently reported catalysts. Chronoamperometry tests were performed under ambient conditions at different potentials.

## Supplementary Material

nwaa213_Supplemental_File
