# Supplementary material for: Graphdiyne-based metal atomic catalysts for synthesizing ammonia
Source: Natl Sci Rev. 2020 Aug 28;8(8):nwaa213. doi: 10.1093/nsr/nwaa213 (PMC8363333; doi:10.1093/nsr/nwaa213)
Supplement: nwaa213_Supplemental_File [file nwaa213_supplemental_file.docx]

Supplementary Information for

Graphdiyne based metal atomic catalysts for synthesizing ammonia

Huidi Yu^1^, Yurui Xue^1,2^*, Lan Hui^1^, Chao Zhang^1^, Yan Fang^1^, Yuxin Liu^1^, Xi Chen^1^, Danyan Zhang^1^, Bolong Huang^3,^* and Yuliang Li^1,4,^*

^1^Institute of Chemistry, Chinese Academy of Sciences, Beijing 100190, P.R. China; ^2^Science Center for Material Creation and Energy Conversion, School of Chemistry and Chemical Engineering, Shandong University, Jinan 250100, P.R. China; ^3^Department of Applied Biology and Chemical Technology, the Hong Kong Polytechnic University, Hung Hom, Kowloon, Hong Kong SAR, 999077, P. R. China and ^4^University of Chinese Academy of Sciences, Beijing 100049, P. R. China

***Corresponding authors.** E-mails: xueyurui@iccas.ac.cn; ylli@iccas.ac.cn; bhuang@polyu.edu.hk

**Treatment of Nafion Membranes.** The Nafion membrane was pre-treated by boiling 1 h in water, 1 h in H_2_O_2_, another 1 h in water, 3 h in 0.5 M H_2_SO_4_ and an additional 6 h in water sequentially. All these treatments were conducted at 80 °C.

**NH_3_ determination.** The electrolyte in cathodic chamber after ECNRR measurements was transferred out, and the Nafion membrane was immersed into above electrolyte overnight for collecting absorbed NH_3_. When tested in 0.1 M Na_2_SO_4_, NH_3_ concentration was determined by a spectrophotometry method. Typically, 50 μL oxidizing solution (NaClO with ρCl=4~4.9 and 0.75 M NaOH), 500 μL coloring solution (0.4 M C_7_H_5_O_3_Na together with 0.32 M NaOH) and 50 μL catalysts solution (1 wt.% Na_2_[Fe(CN)_5_NO]·2H_2_O) were added into 4 mL above post-tested electrolyte in sequence. After standing in ambient condition for 1 h, UV-Vis absorption measurements were performed to the above solution with color regents. From the absorbance at the wavelength of 660 nm, the NH_3_ yield can be quantified according to the calibration curve. The absorbance-concentration curve was calibrated from standard NH_3_ solution (0-1μg/mL), and showed reliable linear relationship (y=0.2051x+0.0143, R^2^=0.998). When tested in 0.1 M HCl, a typical indophenol method was used for the NH_3_ quantification. In brief, 2 mL color solution (5% salicylic acid and 5% sodium citrate in 1 M NaOH), 1 mL oxidizing solution (0.05 mL NaClO), and 200 μL catalysts solution (1 wt.% Na_2_[Fe(CN)_5_NO]·2H_2_O) were mixed with 2 mL above gathered electrolyte. The UV-Vis absorption data at 655 nm were collected after 2 h standing in 25 °C. The calibration curved obtained from standard NH3 solution with a series concentration exhibited good linear relationship (y=0.1485x+0.0056, R^2^=0.999).

For ECNRR in both 0.1 M Na_2_SO_4_ and 0.1 M HCl, the average NH_3_ yield (Y_NH3_) can be calculated (normalized by catalysts mass loading) as equation 1:

Y_NH3_ = (W_NH3_ × V)/(t × m_cat._) (1)

or normalized by geometric surface area using the equation 2:

Y_NH3_ = (C_NH3_ × V)/(t × A) (2)

where W_NH3_ and C_NH3_ is the mass concentration and molar concentration of NH_3_, respectively, V stands for the total volume of electrolyte in cathodic chamber for ECNRR, t is the time for catalytic process, m_cat._ and A represent mass loading and geometric surface area of the catalysts, respectively.

Subsequently, for ECNRR in both conditions, the total consumption of electricity (Q) was obtained from the integration of the current-time curve. Accordingly, Faradic efficiency (FE) can be calculated according to the equation 3 (F is the Faradic Constant):

FE = (3F × C_NH3_ × V)/Q (3)

**Calculation details.** Density functional theory (DFT) calculations based on the CASTEP code has been performed. The on-site Pd-4d orbital projections and decomposition are carried out with self-energy minimization method. Simple rotationally invariant DFT+U framework imbedded within the CASTEP source code. The GDY-Pd with different N-fixation and reduction configurations has been self-consistently performed based on the Broyden-Fletcher-Goldfarb-Shannon (BFGS) geometry optimization algorithm. The PBE exchange and correlation functional is chosen and the plane wave basis set cut-off energy is 750 eV with a Monkhost-Pack k-point mesh of 4×4×2. The ensemble DFT (EDFT) has been used for preventing the spurious spin-charge perturbation for converging electronic minimization. The convergence tolerance of total energy calculation is determined at 5.0 x 10^−7^ eV/atom with ionic force minimization level of 0.001 eV/ Å by Hellmann-Feynman theorem.

The Pd, C, N, and H norm-conserving pseudopotentials are generated using the OPIUM code in the Kleinman-Bylander projector form, and the non-linear partial core correction for the Pd valence electrons and a scalar relativistic averaging scheme are selected to treat the spin-orbital coupling effect. We chose the (4*d,* 5*s*, 5*p*), (2*s*, 2*p*), (1s) states as the valence states of Pd, C, N, and H atoms respectively. The RRKJ method is chosen for the optimization of the pseudopotentials. The Hubbard U on the Pd-4d is self-consistently to be U_d_=7.56 eV.

To understand the optimal N-fixation, we have summarized different N_2_-adsorption energies and corresponding electronic configurations. The subtle interplay between Pd-4d and N_2_-2p orbitals has been illustrated with the projected partial density of states (PDOSs) analysis. These two sets of energetic scales (N_2_-adsorption and orbital potential energy) are essentially helpful to us for excavating the N-fixation variation trend associate with orbital electronic activity.

With the adsorption energy increases, the Pd-4d band center monotonically deepens from E_V_-1.4 eV (E_V_=0 for E_F_­) to E_V_-3.1 eV. The N_2_-p-band widths (bonding, anti-bonding) are also rapidly widened from (3 eV, 6 eV) to (9 eV, 8 eV) and presenting from localized to diffused characters for p-electrons, respectively. Meanwhile, the splitting positions of bonding (N_2_-2pσ) anti-bonding orbital (N_2_-2pσ*) relative to the Pd-4d band center has also indicated. From the different orbital variation trends, we find that the energetic preference of N_2_-adsorption on the GDY-Pd interacting with Pd-4d orbitals indeed follows the farthest p-d separation regulation instead of obeying the well-understood p-d orbital overlapping rule. Such trend means a repulsive Coulomb potential always interfering the p-d orbital coupling. On the other hand, from the view on p-d orbital overlapping, opposite trend of energetic preference comparing the orbital overlapping implies an even stronger p-d orbital correlation favors the electron-transfer to facilitate the N_2_-adsorption on the GDY-Pd system.

As above mentioned, this is evidently different from the adsorption preference of O-species from the catalytic redox reactions. Such anomalous orbital variation trend implies the on-site effective screening Coulomb repulsion potential is a determining factor existing between Pd-4d^10^ and N_2_-(2s^2^, 2p^3^). This arises because the non-bonding lone-pair electrons are sourced from totally different orbital type. For the O-species related catalysis, the long-pair electrons come from the O-2p orbital, which are also active in p-d orbital overlapping induced electron-transfer from surface bonding during the chemisorption. However, on the contrast, long-pair electrons from N_2_-2s-orbitals are highly active participating within ECNRR process for hydrogenation-bonding but repulsive mediating between p- and d- orbitals.

The fulfilled Pd-4d^10^ is electron-rich center for evident site-to-site charge migrations exhibiting substantially strong electronegative activity (ENA). Such strong ENA induces high chemical potential contrast for favorite directional electron-transfer, which means the GDY-Pd favors electron-transfer from Pd onto N-species for more efficient N-hydrogenation within ECNRR process instead of proton-electron charge exchange for HER. We further interpret the underlying electronic mechanism for redirect HER-suppression trend. The p-d coupled effective negative correlation energy matters the subtle interplay between on-site Coulomb repulsion and ENA, which overcomes on-site Coulomb repulsion and being energetically favorable to accumulate ENA.


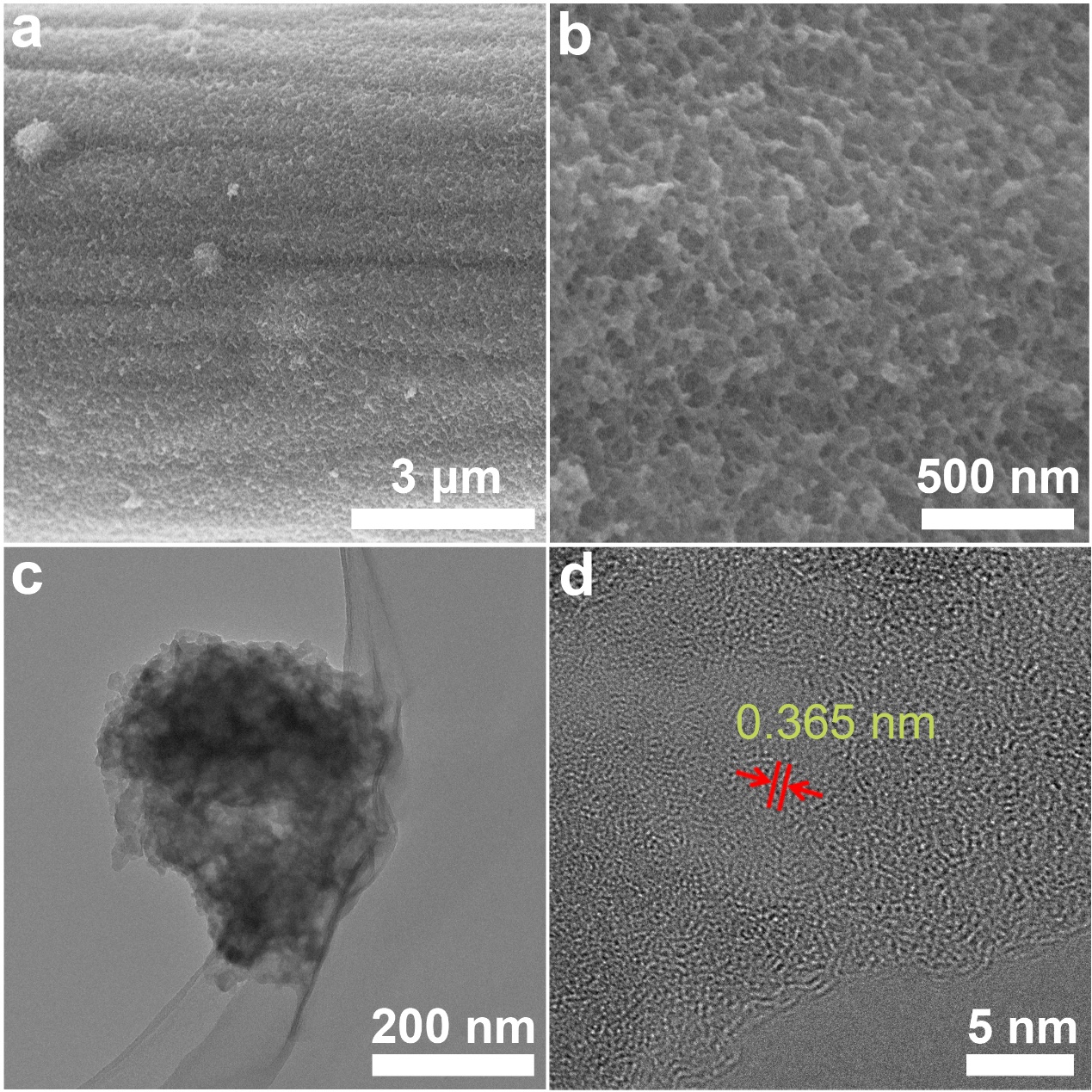


Supplementary Figure S1. (a) Low- and (b) high-magnification SEM, (c) TEM and (d) HRTEM images of GDY.


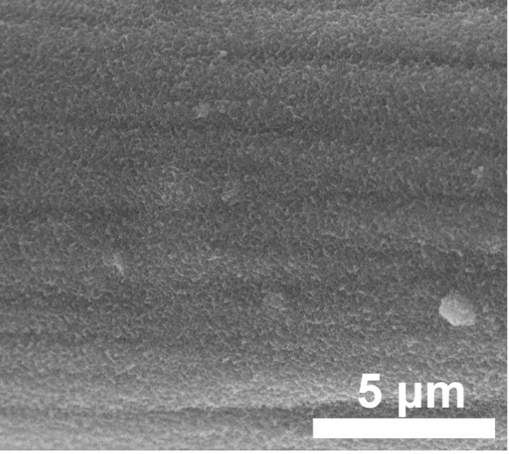


Supplementary Figure S2. SEM image of Pd-GDY.


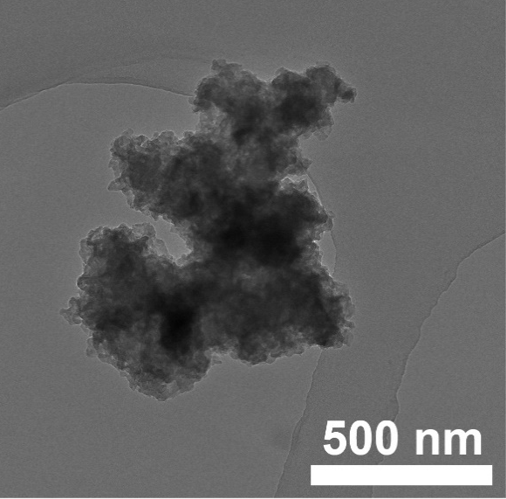


Supplementary Figure S3. TEM image of Pd-GDY.


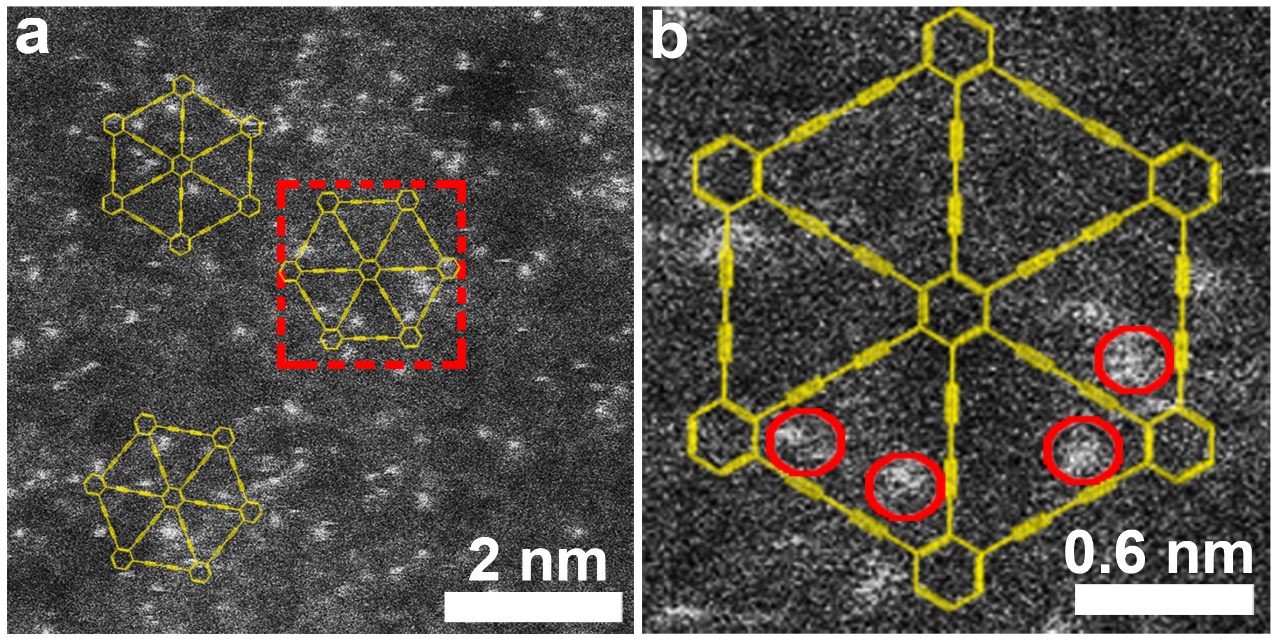


Supplementary Figure S4. (a,b) The HAADF images of Pd-GDY in different magnification.





Supplementary Figure S5. Histogram for the size distribution of Pd atoms.

**
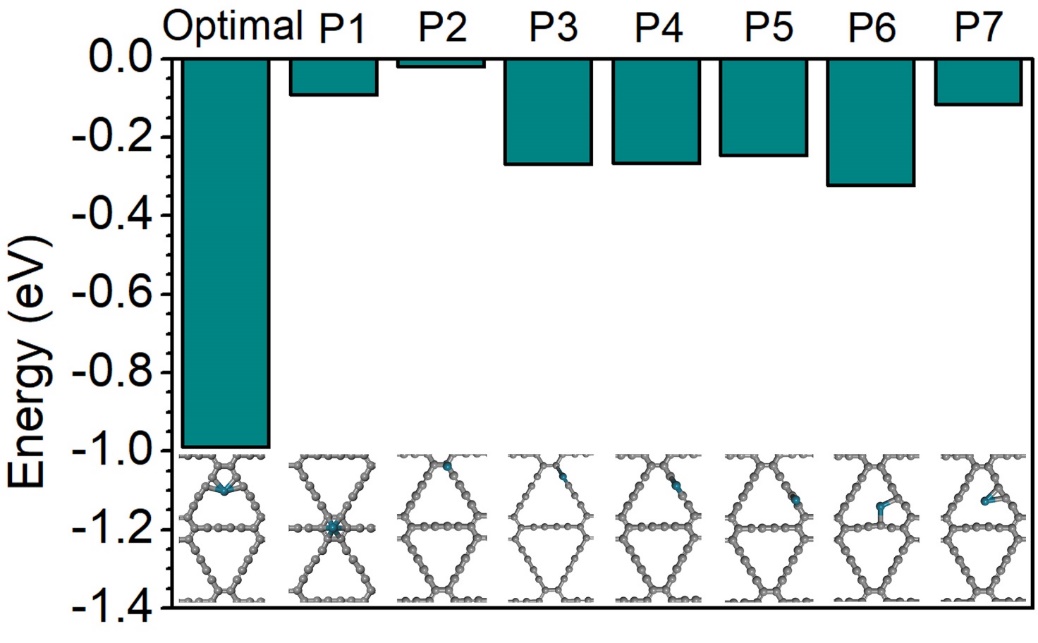
**

Supplementary Figure S6. The formation energies comparison of different anchoring sites on different sites.

All the possible placements of the Pd on GDY have been fully examined. The anchoring site is determined based on the formation energies comparison between different anchoring sites. Notably, the lowest formation energy of the anchoring site of -0.99 eV as the presented position in Fig. 2 (in main text) will be the undoubtfully optimal placement for Pd on GDY. In comparison, most other placements for Pd on the GDY (P1-P7) will requires at least two times higher formation energies, indicating the unstable anchoring sites. Thus, the most preferred anchoring position of Pd on GDY has been determined as presented of Fig. 2 in main text.


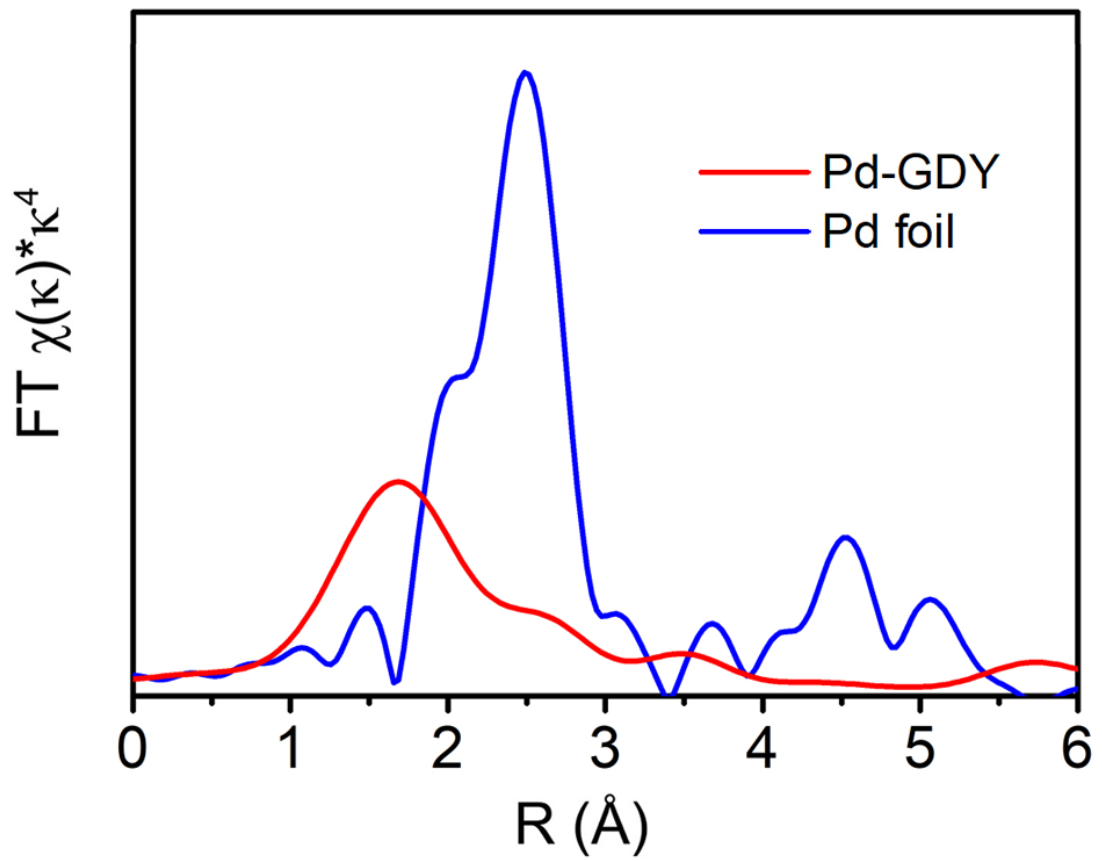


Supplementary Figure S7. FT-EXAFS spectra of Pd-GDY (red line) and Pd foil (blue line) at the Pd K-edge.


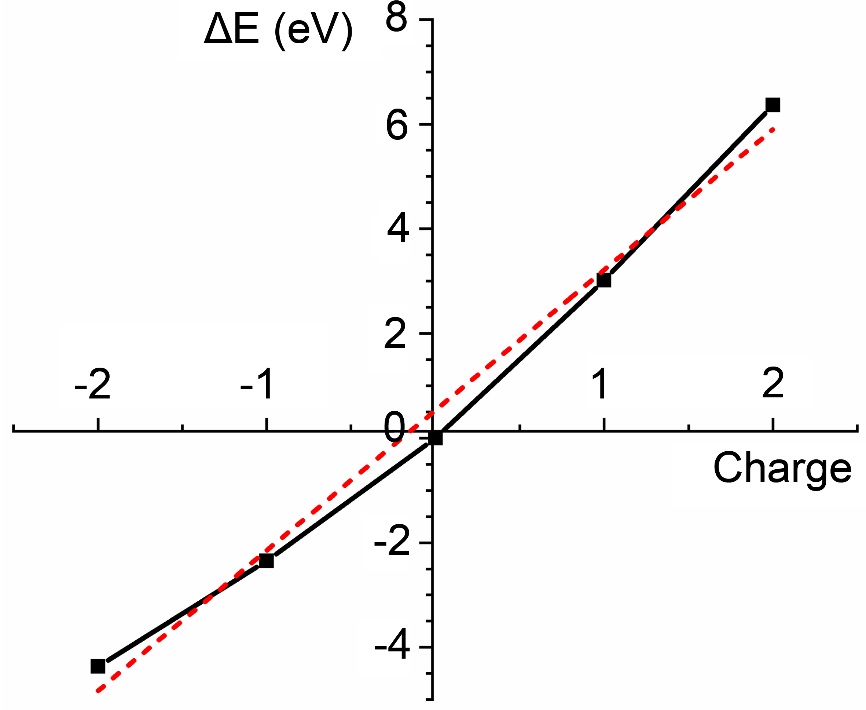


Supplementary Figure S8. The electron-affinity determination of Pd on GDY.

We want to clarify that the zero-valence Pd on the surface did not represent the absence of electron transfer that leads to weak interaction with the substrate. Instead, the zero-valence will be considered as the consequence of the active electron transfer between the Pd and the GDY rather beyond the conventionally identified covalent bond or ionic bond between Pd and C. In our previous work, a similar phenomenon is also reported in other transition metal as Ni (See ***Nat. Commun.***, 2018, **9**, 1460). The zero-valence of the anchoring metal is achieved by the abnormal strong (*sp*)-*d* overlapping, which will induce inert closed-shell effect for transition metal on GDY and lead to an extrinsic charge compensation to reduce the oxidation state of metal to the zero valence state. As for Pd with a 4d^10^ electron configuration, the relatively inert reactivity will be activated to strong electron-affinity based on the local d-d coupling, which will enhance the electron transfer ability for efficient ECNRR. the extra active electrons from local GDY contribute to the formation of an electron-affinitive 4d^10+δ^ Pd, which can break the constraints of electron supply to achieve the firm adsorption of N_2_ to initiate the ECNRR. Following the previously reported approach in calculating the orbital energy, we are able to confirm that the Pd shows a stronger electron-affinity with a large δ value, supporting the higher activity in electron transfer. The intercept of the linear fitting has determined the δ value of Pd will be 0.5337. Meanwhile, the large slope of the fitting demonstrates high stability of zero-valence rather than being oxidized due to the high energy barrier of losing electrons. Therefore, here in this work, we believe that the Pd will keep zero-valence based on the strong interaction with the GDY substrate.

**
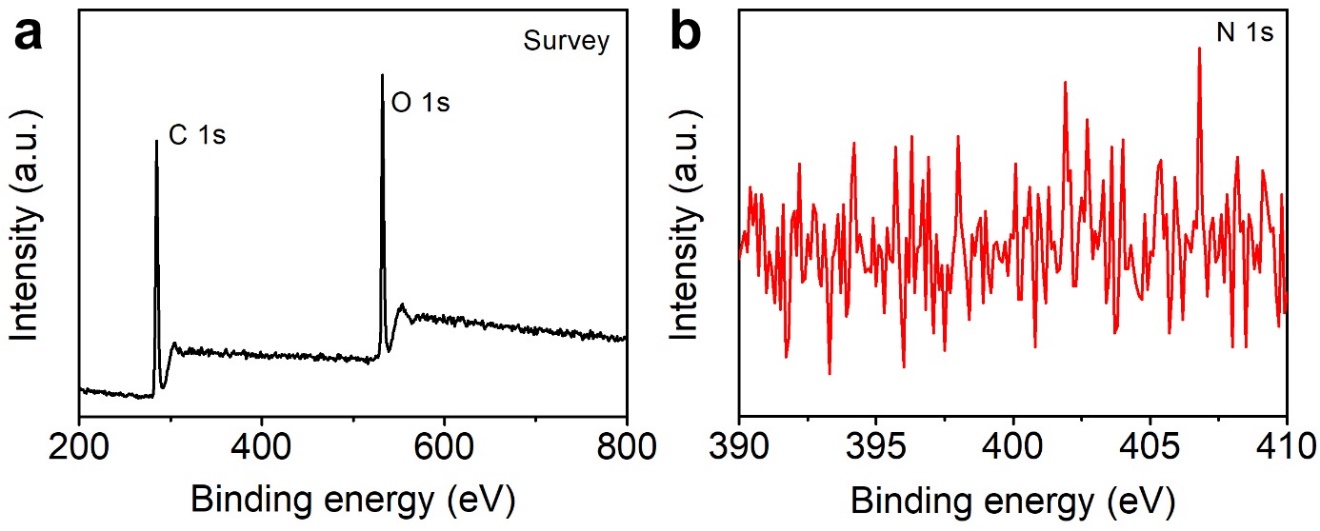
**

Supplementary Figure S9. (a) XPS survey and (b) N 1s XPS spectra of Pd-GDY. No N peak can be observed from the XPS survey spectrum.


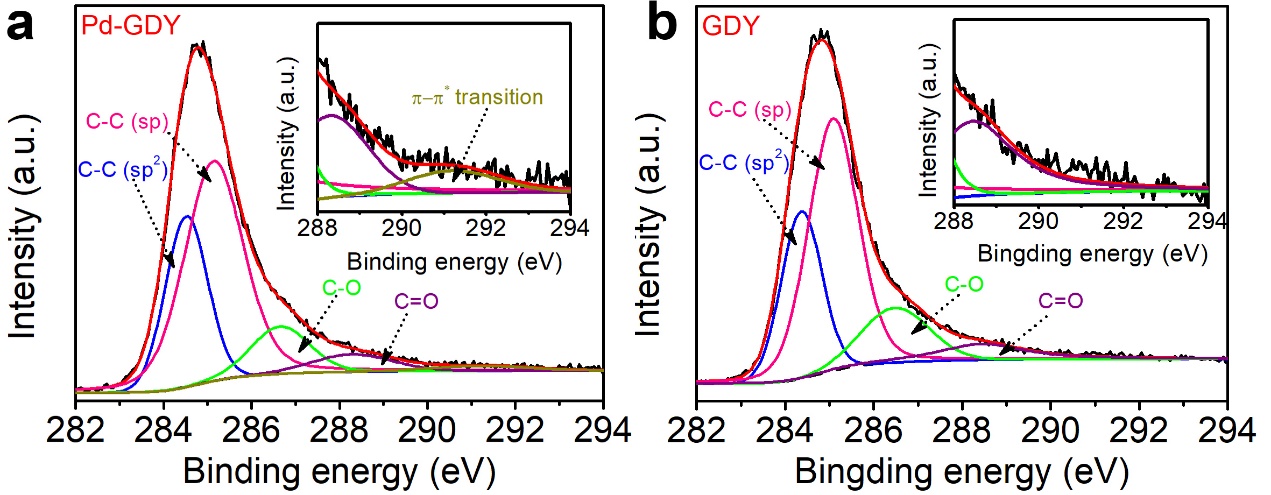


Supplementary Figure S10. XPS C 1s spectra of (a) Pd-GDY and (b) GDY.

The area ratio of sp-C and sp^2^-C in both Pd-GDY and pristine GDY is 2, demonstrating the anchoring of Pd atoms does not destruct the chemical structure of GDY network. The inset of (a) shows the additionally denconvoluted peak at 291.2 eV, which originated from the π-π* transition between GDY and anchored Pd atoms.


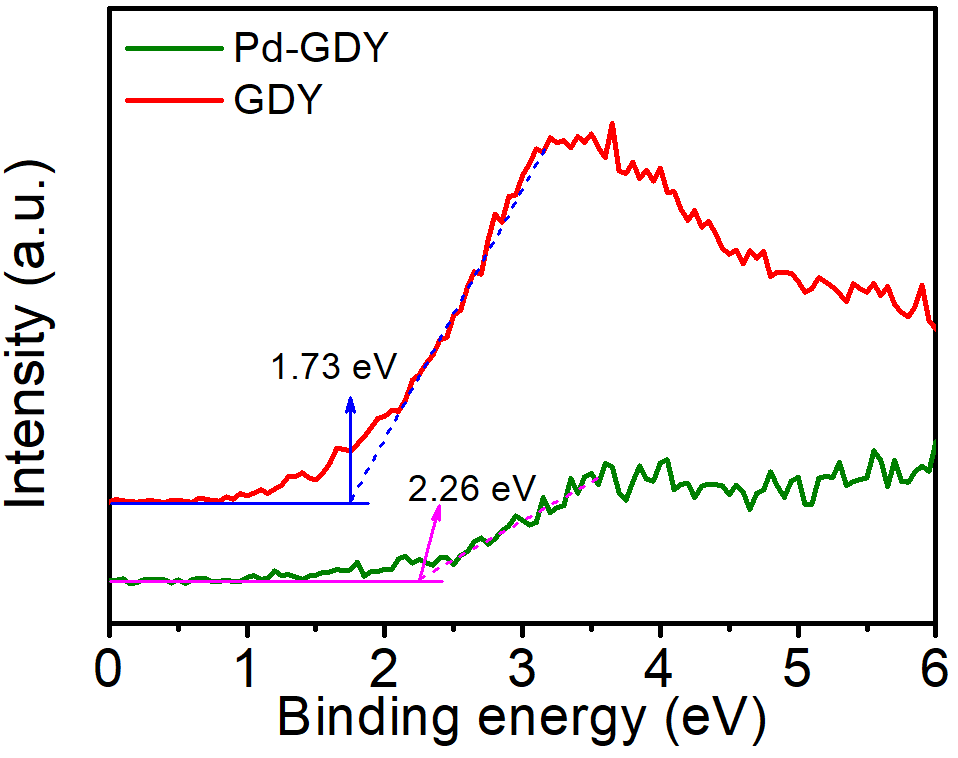


Supplementary Figure S11. Valence band spectra of Pd-GDY (red line) and GDY (green line).


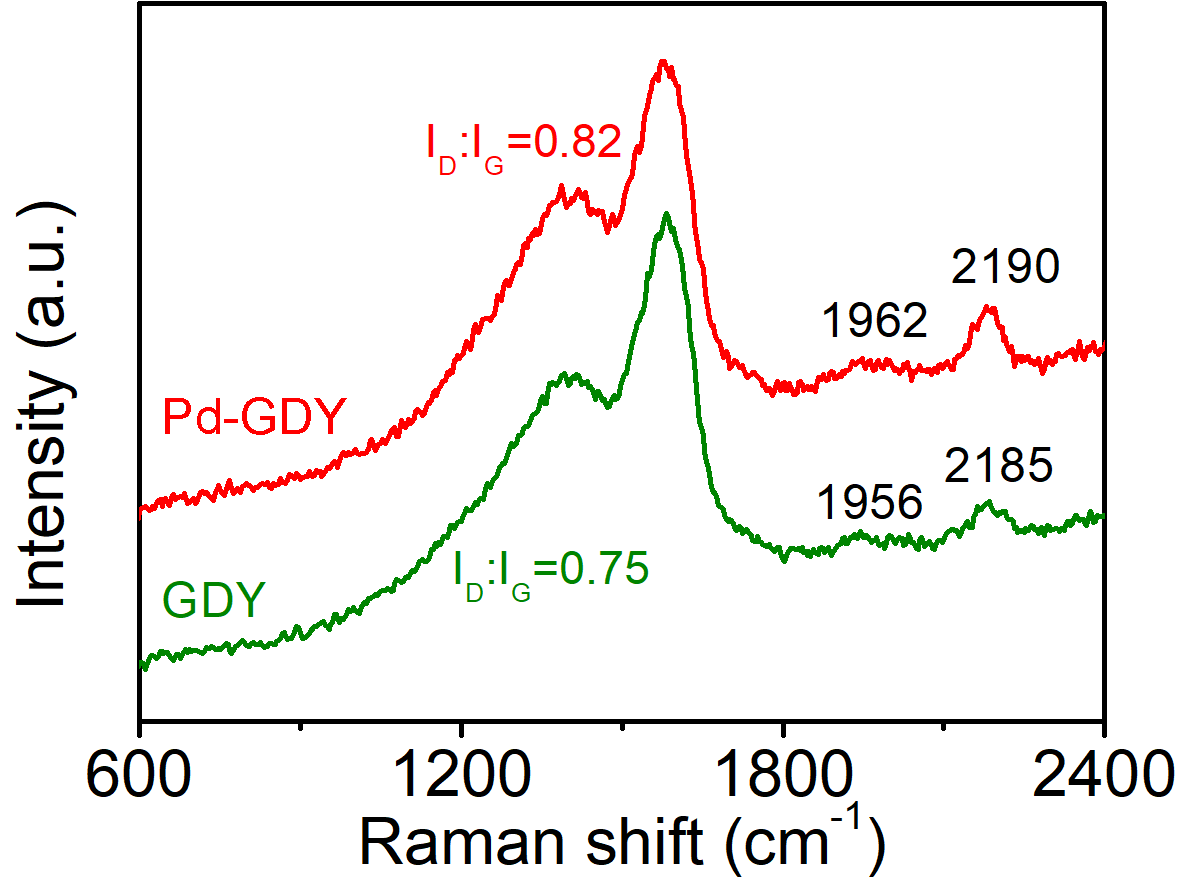


Supplementary Figure S12. Raman spectra of Pd-GDY (red line) and GDY (green line).

**
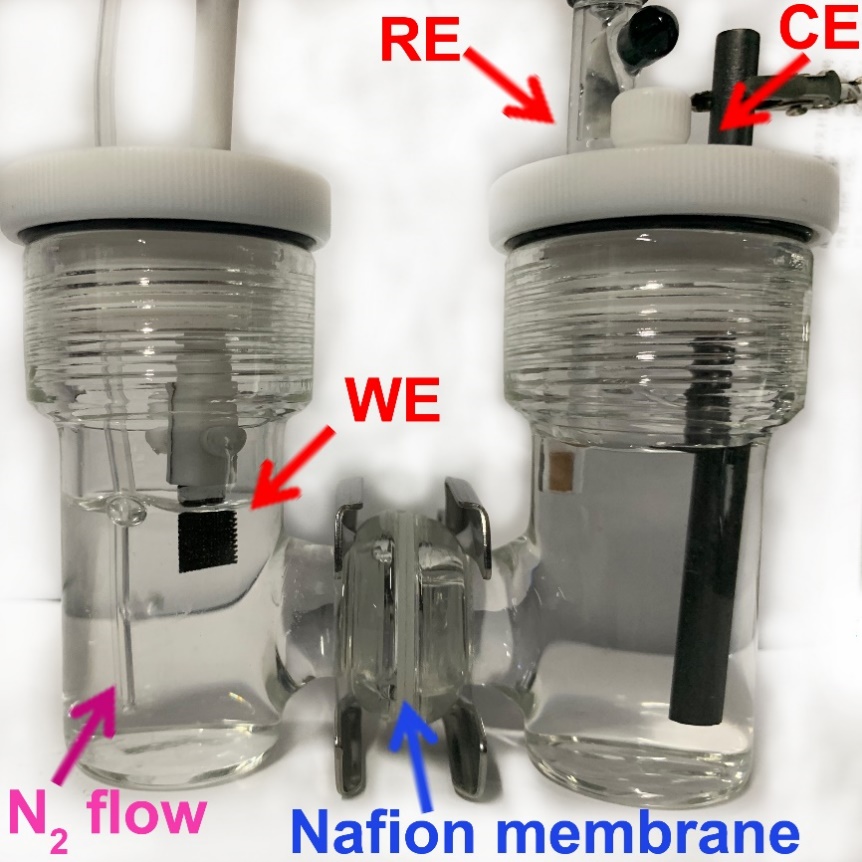
**

Supplementary Figure S13. Optical photograph of ECNRR reactor.

**
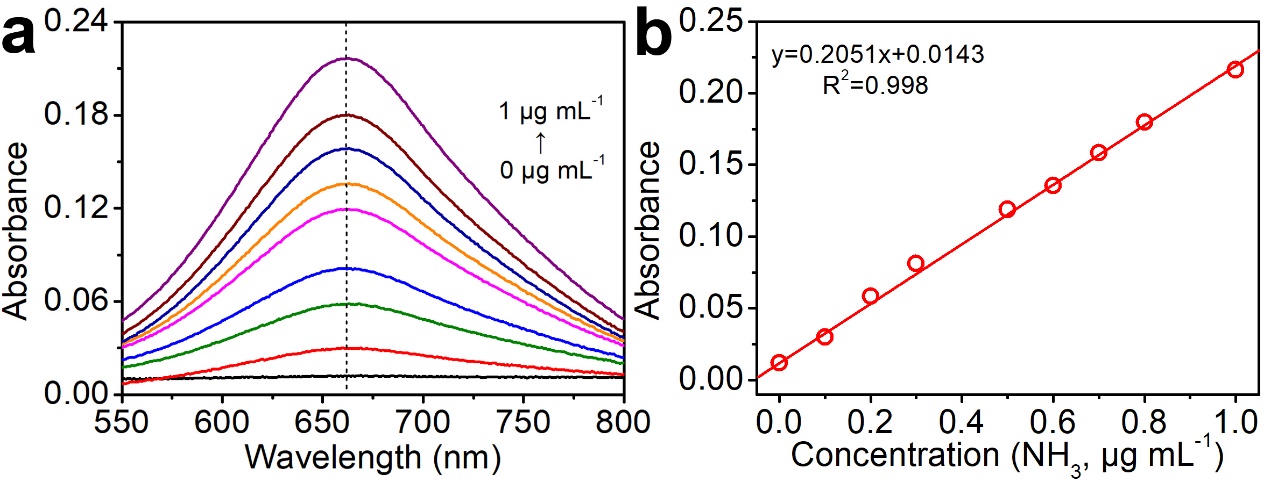
**

Supplementary Figure S14. (a) UV-Vis absorption spectra of indophenol assays with standard NH_3_ solution after incubated for 1 h at room temperature. (b) Corresponding Calibration curve.


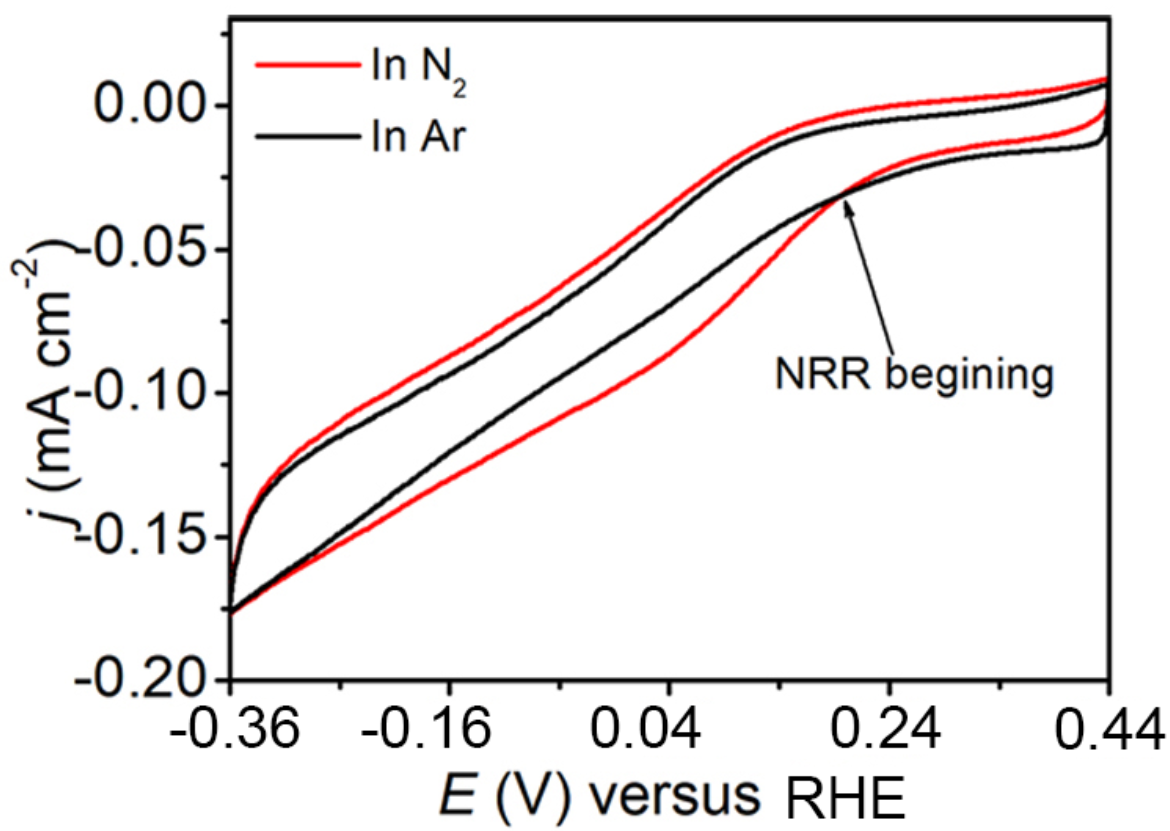


Supplementary Figure S15. Cyclic voltammetry curves of Pd-GDY obtained in N2- and Ar-saturated electrolyte.


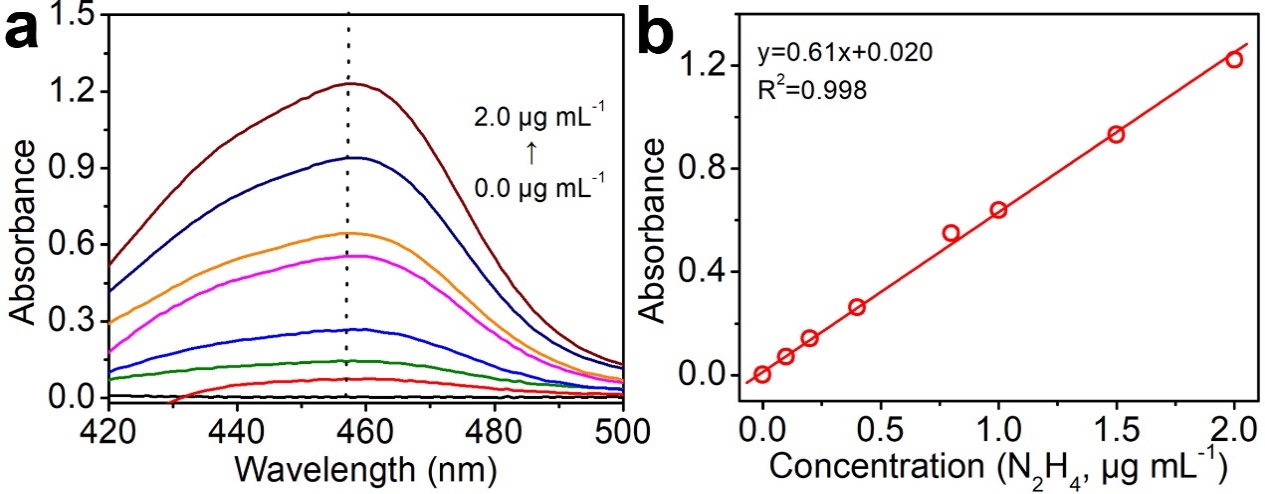


Supplementary Figure S16. (a) UV-Vis absorption spectra of standard N_2_H_4_ solution with various concentration after incubated for 10 min at room temperature in 0.1 M Na_2_SO_4_. (b) Corresponding calibration curve.


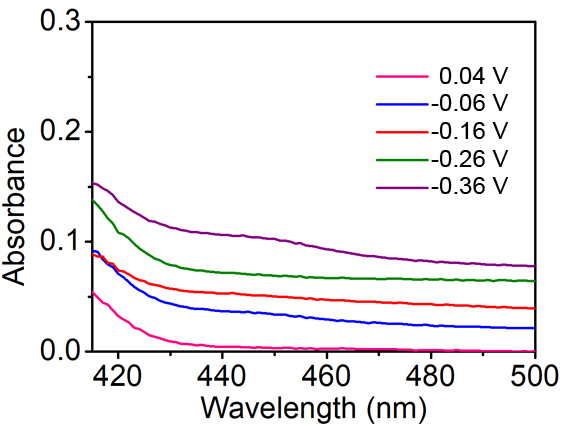


Supplementary Figure S17. Detection of N_2_H_4_ obtained in 0.1 M Na_2_SO_4_ at different applied potentials.

Almost no absorbance peak appeared at 455 nm, demonstrating the negligible N_2_ conversion to N_2_H_4_.

**
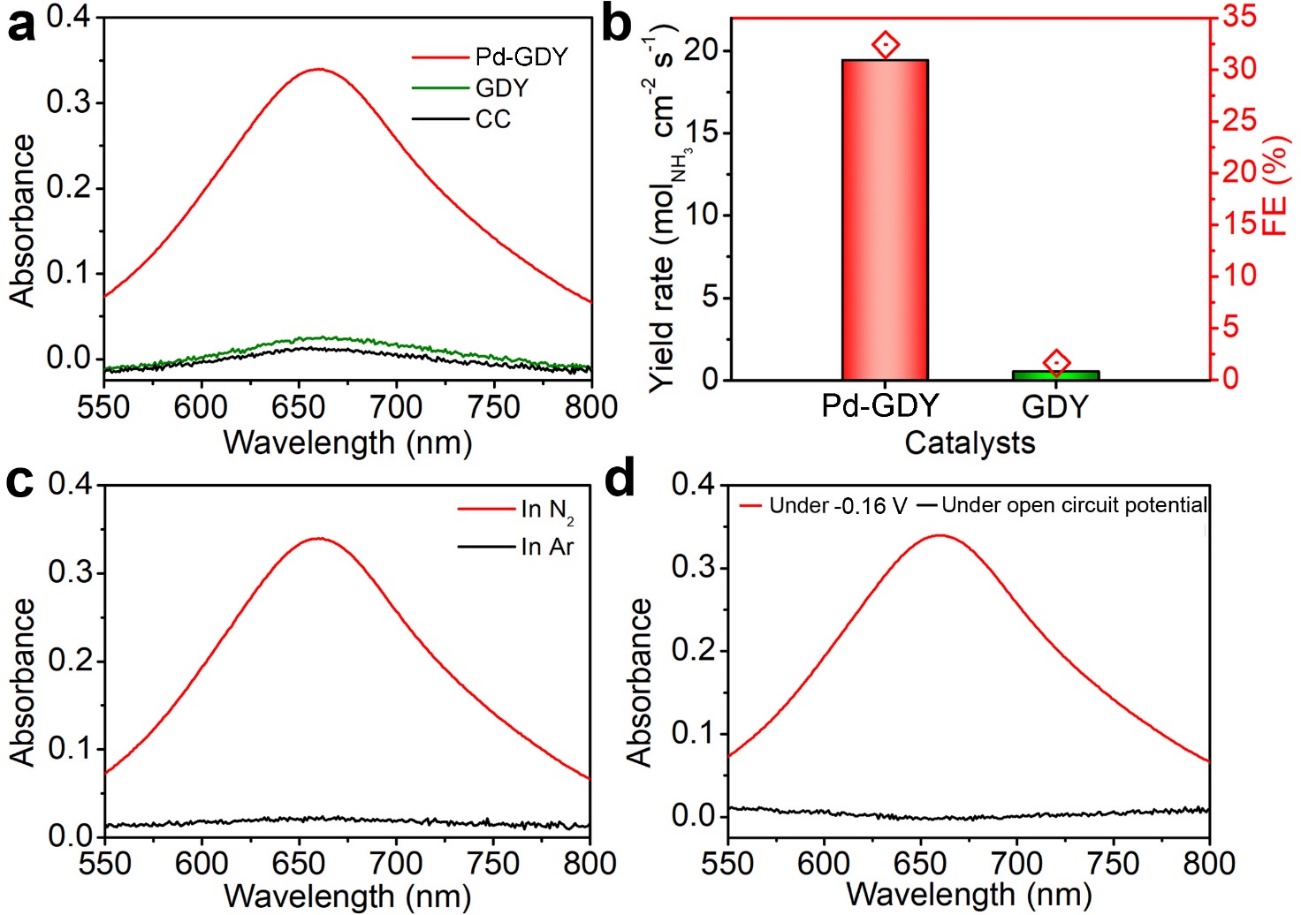
**

Supplementary Figure S18. (a) UV-Vis absorption spectra of Pd-GDY, GDY and CC after ECNRR at -0.16 V. (b) The Y_NH3_ and FE of Pd-GDY and pristine GDY at -0.16 V. (c) UV-Vis absorption spectra of Pd-GDY after 2 h electrocatalysis in N_2_ and Ar atmosphere at -0.16 V and stained with indophenol assays for 1 h. (d) UV-Vis absorption results of Pd-GDY toward electrocatalysis process at -0.16 V and open circuit potential.


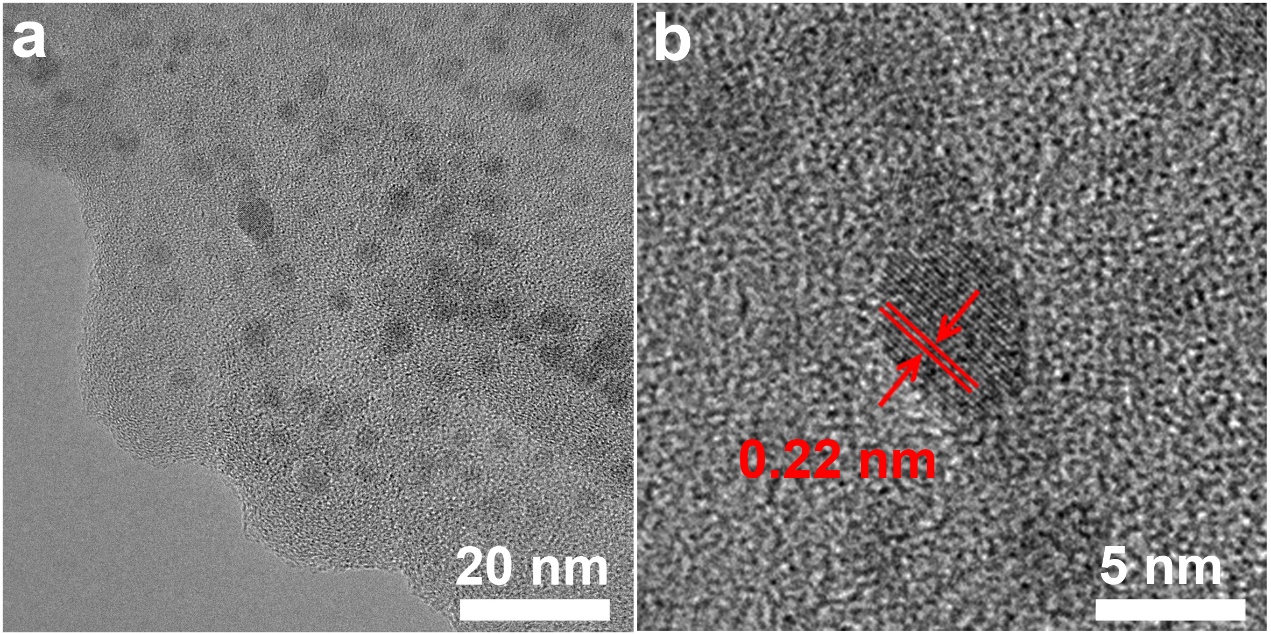


Supplementary Figure S19. (a) TEM and (b) HRTEM images of Pd NP-GDY.

Pd nanoparticles were uniformly distributed on GDY. The lattice fringe of (111) face of metallic Pd (0.22 nm) was observed, demonstrating the successful preparation of Pd NP-GDY.

**
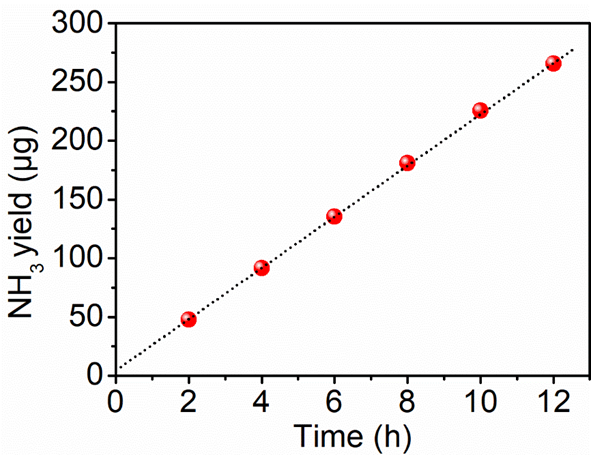
**

Supplementary Figure S20. Total NH_3_ yield against reaction time.


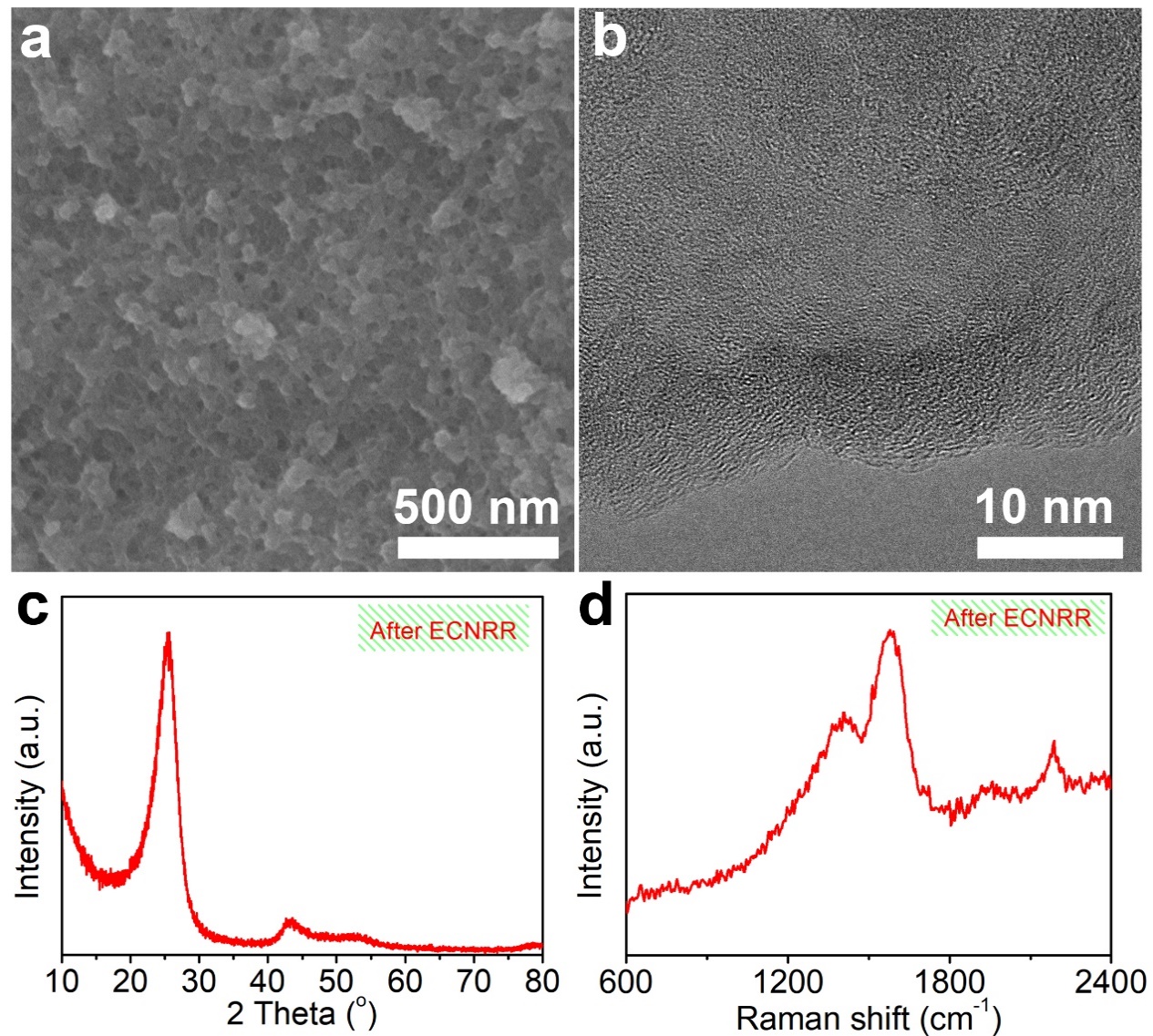


Supplementary Figure S21. (a) SEM image, (b) HRTEM image, (c) XRD pattern, and (d) Raman spectrum of Pd-GDY after 6 cycles ECNRR in 0.1 M Na_2_SO_4_.


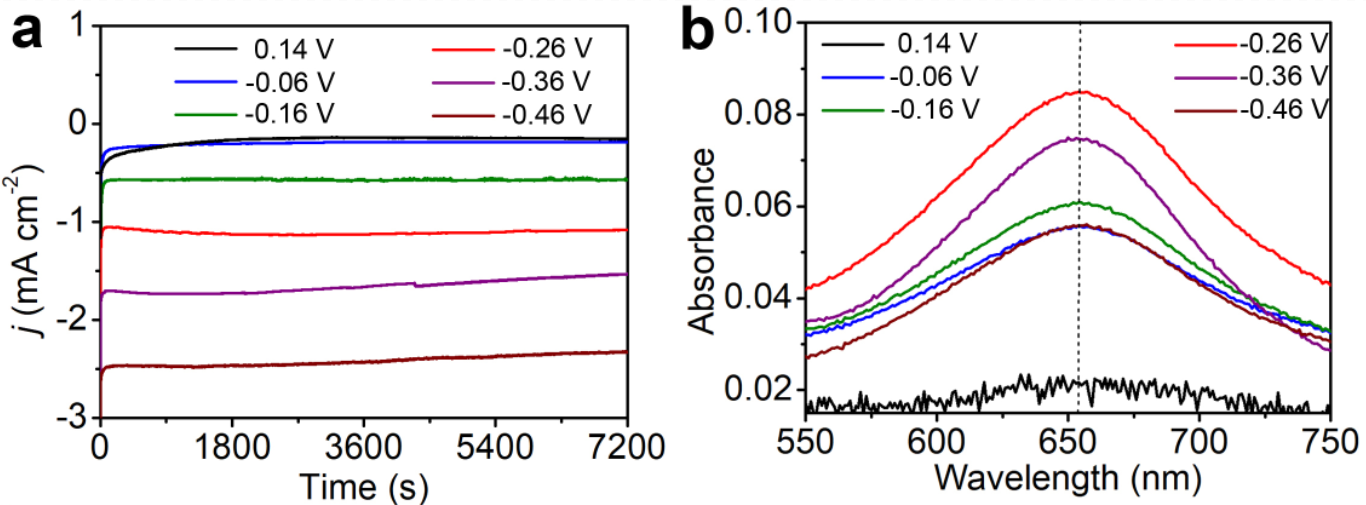


Supplementary Figure S22. (a) Time-current density curves of Pd-GDY at different applied potentials. (b) UV-Vis absorption spectra of the 0.1 M HCl electrolytes after ECNRR at different potentials for 2 h.


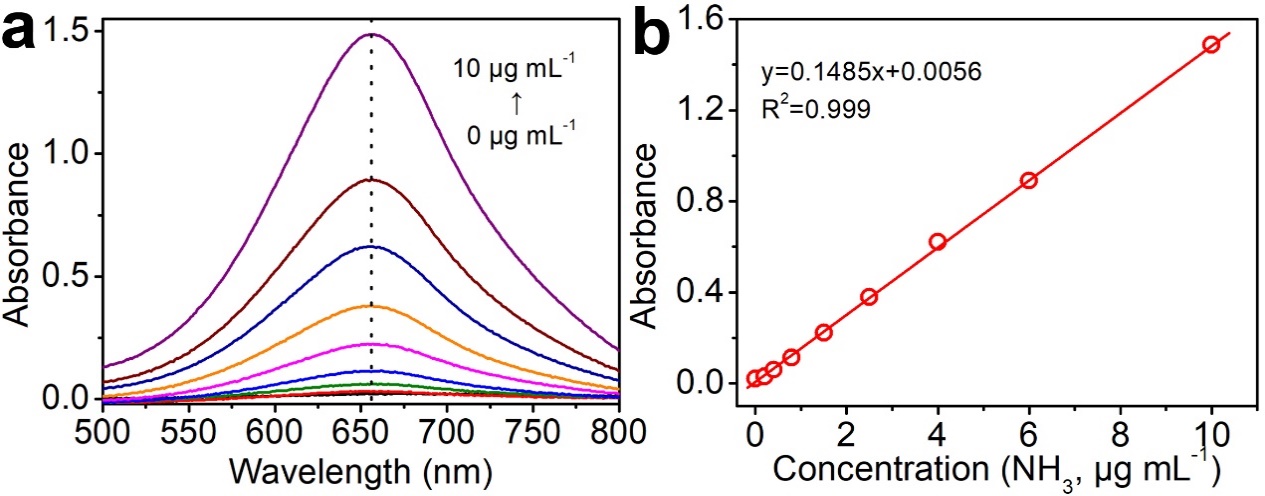


Supplementary Figure S23. (a) UV-Vis absorption spectra of standard NH_3_ solution with indophenol assays after standing 2 h at ambient atmosphere. (b) Corresponding calibration curve.


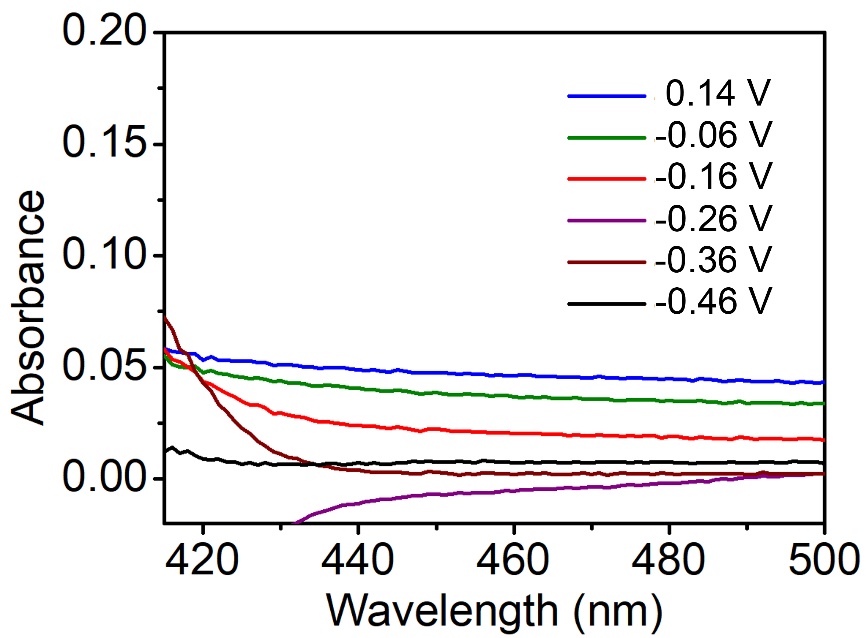


Supplementary Figure S24. N_2_H_4_ detection in 0.1 M HCl after ECNRR at different potentials.


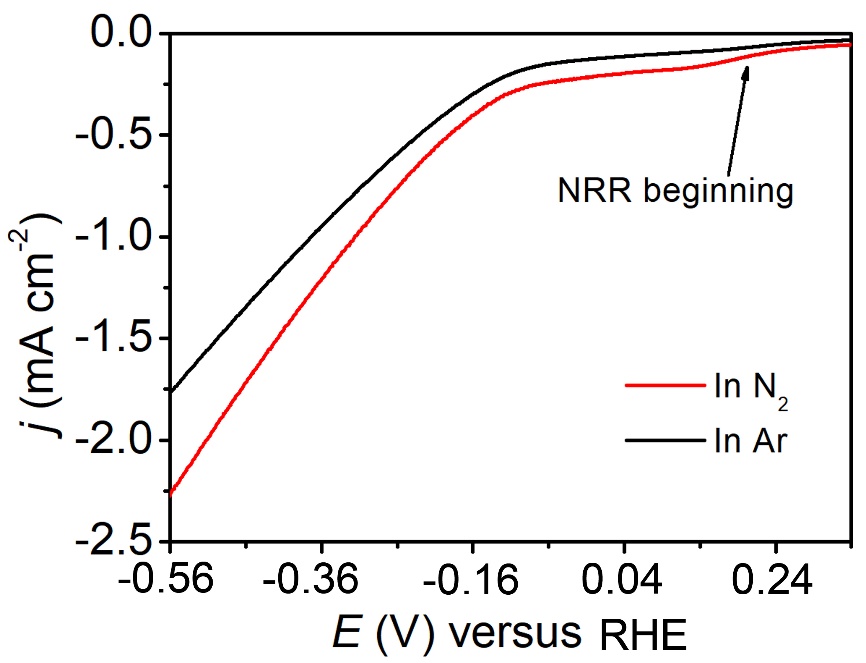


Supplementary Figure S25. Polarization curves of Pd-GDY in N_2_– and Ar–saturated 0.1 M HCl.

The additional reduction peak appeared at approximate 0.2 V in N_2_ suggested the beginning of ECNRR. The rapidly increasing current density after around -0.16 V indicated HER became dominant in both conditions.


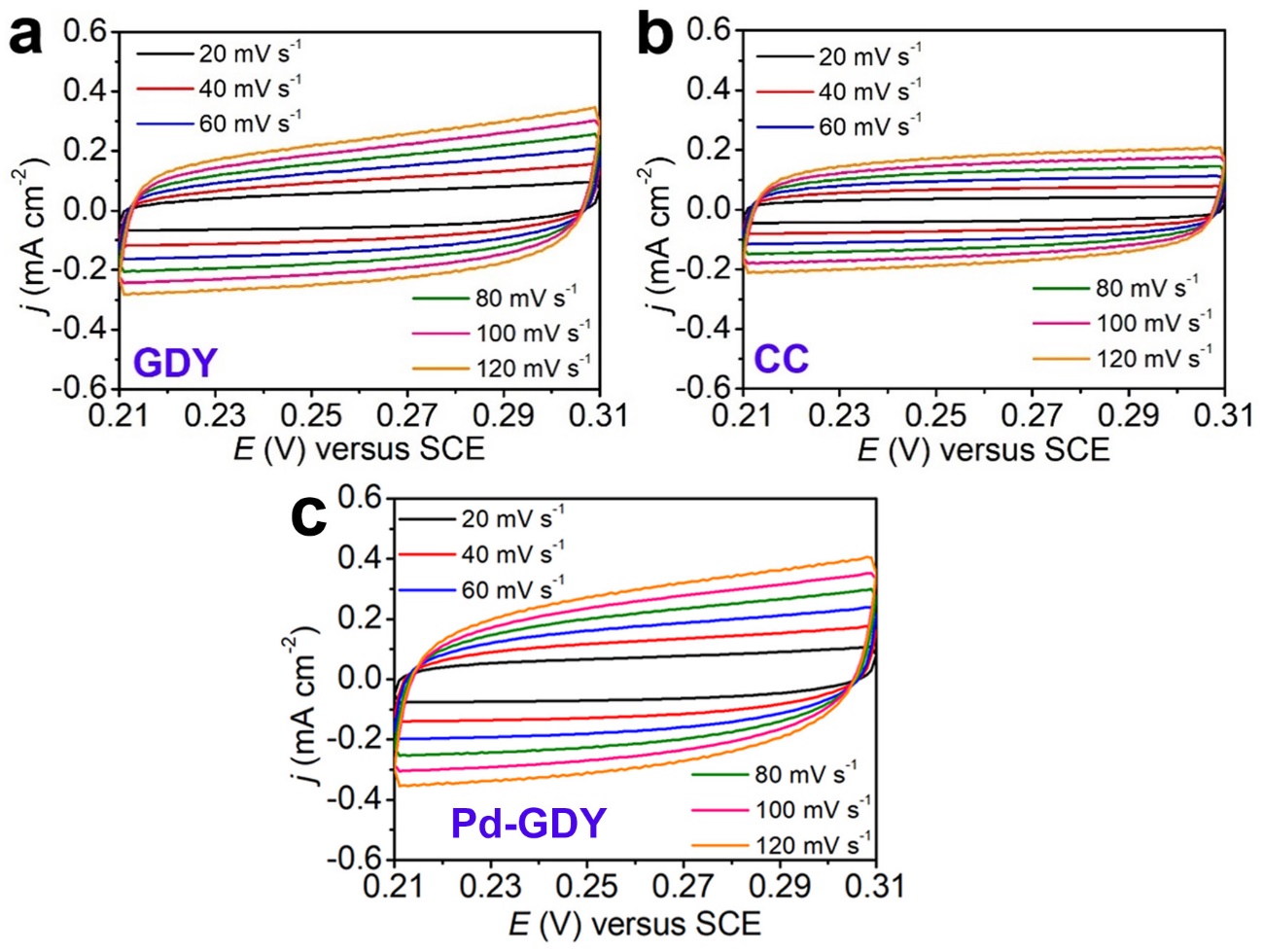


Supplementary Figure S26. CV curves of (a) GDY, (b) CC and (c) Pd-GDY obtained in 0.1 M Na_2_SO_4_.


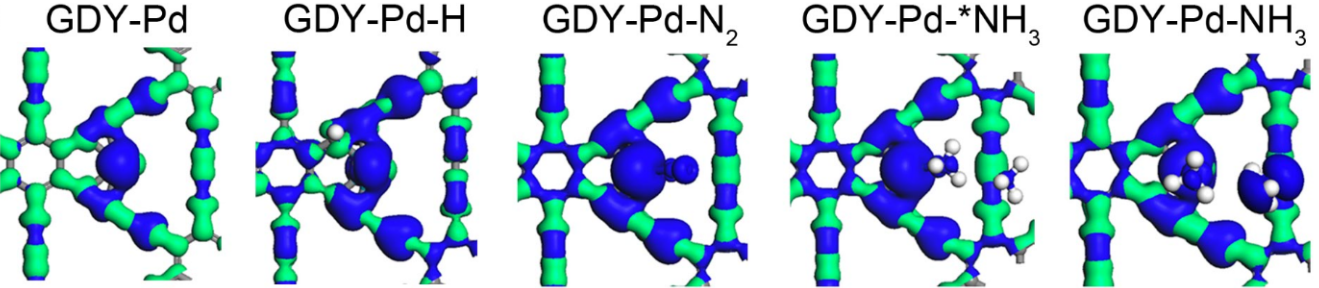


Supplementary Figure S27. The real spatial contour plots for bonding and anti-bonding orbitals near EF for GDY-Pd.

Supplementary Table S1. Y_NH3_ and FE of the ECNRR in 0.1 M Na_2_SO_4_ and 0.1 M HCl under different potentials.

| **0.1 M Na_2_SO_4_** | | |
| --- | --- | --- |
| **Potential** | **Y_NH3_ (mg_NH3_ mg_Pd_ h^-1^)** | **FE (%)** |
| 0.04 | 0.93 ± 0.06 | 16.55 ±0.49 |
| -0.06 | 3.00 ± 0.19 | 23.22 ± 1.32 |
| -0.16 | 4.45 ± 0.30 | 31.62 ± 1.06 |
| -0.26 | 2.74 ± 0.15 | 11.55 ± 2.09 |
| -0.36 | 1.98 ± 0.10 | 9.62 ± 0.94 |
| **0.1 M HCl** | | |
| **Potential** | **Y_NH3_ (mg_NH3_ mg_Pd_ h^-1^)** | **FE (%)** |
| 0.14 | 0.25 ± 0.04 | 1.89 ± 0.25 |
| -0.06 | 0.67 ± 0.08 | 4.32 ± 0.49 |
| -0.16 | 1.10 ± 0.14 | 2.49 ± 0.31 |
| -0.26 | 1.58 ± 0.05 | 1.78 ± 0.08 |
| -0.36 | 1.00 ± 0.05 | 0.79 ± 0.09 |
| -0.46 | 0.87 ± 0.09 | 0.49 ± 0.08 |

Supplementary Table S2. Comparing the ECNRR performances (with Y_NH3_ normalized by catalyst loading) of Pd-GDY with other reported ones.

| **Materials** | **Electrolytes** | **Y_NH3_ (mg_NH3_ mg_cat._^-1^ h^-1^)** | **FE (%)** | **References** |
| --- | --- | --- | --- | --- |
| Pd-GDY | 0.1 M Na_2_SO_4_ | 4.45 | 31.6 | This work |
|  | 0.1 M HCl | 1.58 | 4.32 |  |
| a-Au/CeO_x_–RGO | pH=1 HCl solution | 0.0083 | 10.10 | *Adv. Mater.,* **2017**, *29*, 1700001 |
| Bi_4_V_2_O_11_/CeO_2_ | 0.1 M HCl | 0.02321 | 10.16 | *Angew. Chem. Int. Ed.***, 2018**, *130*, 1–5 |
| Pd_0.2_Cu_0.8_/rGO | 0.1 M KOH | 0.00166 | 4.5 | *Adv. Energy Mater.*, **2018**, *8*, 1800124 |
| N-doped porous carbon | 0.05 M H_2_SO_4_ | 0.0238 | 1.42 | *ACS Catal.*, **2018**, *8*, 1186−1191 |
| Au/TiO_2_ | pH = 1 HCl solution | 0.0214 | 8.11 | *Adv. Mater.*, **2017**, *29*, 1606550 |
| B_4_C/CPE | 0.1 M HCl | 0.02657 | 15.95 | *Nat. Commun.*, **2018**, *9*, 3485 |
|  | 0.1 M Na_2_SO_4_ | 0.0047 | 9.24 |  |
| polymeric carbon nitride | 0.1 M HCl | 0.00808 | 11.59 | *Angew. Chem. Int. Ed.*, **2018**, *57*, 10246–10250 |
| MoS_2_ Nanoﬂower | 0.1 M Na_2_SO_4_ | 0.02928 | 8.34 | *Adv. Energy Mater.*, **2018**, *8*, 1801357 |
| Ru SAs/N-C | 0.05 M H_2_SO_4_ | 0.1209 | 29.6 | *Adv. Mater.*, **2018**, *30*, 1803498 |
| Ru/NC | 0.1 M HCl | 3.6 | 21 | *Chem*, **2018**, *5*, 1 |

Supplementary Table S3. ECNRR performance (with Y_NH3_ normalized by geometric surface area) of Pd-GDY and other catalysts in various systems.

| **Materials** | **Electrolytes** | **Y_NH3_**  **(×10^-11^ mol_NH3_ cm_cat._^-2^ s^-1^)** | **FE (%)** | **References** |
| --- | --- | --- | --- | --- |
| Pd-GDY | 0.1 M Na_2_SO_4_ | 19.7 | 31.6 | This work |
|  | 0.1 M HCl | 6.99 | 4.32 |  |
| PEBCD/C | 0.5 M Li_2_SO_4_ | 3.28 | 1.71 | *J. Am. Chem. Soc.*, **2017**, *139,* 9771−9774 |
| Fe_2_O_3_-CNT | diluted KHCO_3_ aqueous solution | 0.359 | 0.15 | *Angew. Chem. Int. Ed.,* **2017**, *56,* 2699–2703 |
| Ag-Au@ZIF | THF-based solution | cat. 1 | 18 | *Sci. Adv.*, **2018**, *4*, eaar3208 |
| Fe_2_O_3_ | Molten hydroxide (250 °C/25 bar) | 35% N_2_ conversion rate |  | *Science,*. 2014, *345*, 637 |
| Pd | Perovskite-type solid electrolyte SrCe_0.95_Yb_0.05_O_3_ (SCY), 570 °C | 450 |  | *Science,*. 1998, *282*, 98 |
| MoS_2_ | 0.1 M Na_2_SO_4_ | 8.08 | 1.17 | *Adv. Mater.*, 2018, *30*, 1800191 |
|  | 0.1 M HCl | 8.48 | 0.096 |  |
| vanadium nitride nanoparticles | Nafion | 33 | 6 | *J. Am. Chem. Soc.*, **2018**, *140*, 13387−13391 |
| NCM-Au NP | 0.1 M HCl | 13.07 | 5.2 | *Angew. Chem. Int. Ed.*, **2018**, *57*,12360–12364 |
| Ag nanosheets | 0.1 M HCl | 4.62 | 4.8 | *Chem. Commun.*, **2018**, *54*, 11427–11430 |
| Fe_3_O_4_/Ti | 0.1 M Na_2_SO_4_ | 5.6 | 2.6 | *Nanoscale*, **2018**, *10*, 14386–14389 |

Supplementary Table S4. EIS parameters obtained from R(QR)(QR) equivalent circuit model analysis.

| **Catalysts** | **Pd-GDY** | **GDY** | **CC** |
| --- | --- | --- | --- |
| R_s_ (Ω) | 10.41 | 15.7 | 26.5 |
| CPE_1_ S sec^n^ | 7.747×10^-4^ | 7.817×10^-4^ | 6.302×10^-4^ |
| Freq power, n_1_ (0<n<1) | 0.9188 | 0.9251 | 0.9271 |
| R_ct_ (Ω) | 3501 | 4658 | 8933 |
| CPE_2_ S sec^n^ | 0.07867 | 0.06411 | 0.002382 |
| Freq power, n_2_ (0<n<1) | 0.05803 | 0.07078 | 0.6431 |
| R_1_ (Ω) | 3.481×10^4^ | 3.012×10^5^ | 3198 |
